# Evaluation of drought resistance and transcriptome analysis for the identification of drought-responsive genes in *Iris germanica*

**DOI:** 10.1038/s41598-021-95633-z

**Published:** 2021-08-11

**Authors:** Jingwei Zhang, Dazhuang Huang, Xiaojie Zhao, Man Zhang

**Affiliations:** 1 College of Landscape Architecture and Tourism, Hebei Agricultural University, Baoding, China; 2 State Key Laboratory of North China Crop Improvement and Regulation, Hebei Agricultural University, Baoding, China

**Keywords:** Drought, Plant stress responses

## Abstract

*Iris germanica*, a species with very high ornamental value, exhibits the strongest drought resistance among the species in the genus *Iris*, but the molecular mechanism underlying its drought resistance has not been evaluated. To investigate the gene expression profile changes exhibited by high-drought-resistant *I. germanica* under drought stress, 10 cultivars with excellent characteristics were included in pot experiments under drought stress conditions, and the changes in the chlorophyll (Chl) content, plasma membrane relative permeability (RP), and superoxide dismutase (SOD), malondialdehyde (MDA), free proline (Pro), and soluble protein (SP) levels in leaves were compared among these cultivars. Based on their drought-resistance performance, the 10 cultivars were ordered as follows: ‘Little Dream’ > ‘Music Box’ > ‘X’Brassie’ > ‘Blood Stone’ > ‘Cherry Garden’ > ‘Memory of Harvest’ > ‘Immortality’ > ‘White and Gold’ > ‘Tantara’ > ‘Clarence’. Using the high-drought-resistant cultivar ‘Little Dream’ as the experimental material, cDNA libraries from leaves and rhizomes treated for 0, 6, 12, 24, and 48 h with 20% polyethylene glycol (PEG)-6000 to simulate a drought environment were sequenced using the Illumina sequencing platform. We obtained 1, 976, 033 transcripts and 743, 982 unigenes (mean length of 716 bp) through a hierarchical clustering analysis of the resulting transcriptome data. The unigenes were compared against the Nr, Nt, Pfam, KOG/COG, Swiss-Prot, KEGG, and gene ontology (GO) databases for functional annotation, and the gene expression levels in leaves and rhizomes were compared between the 20% PEG-6000 stress treated (6, 12, 24, and 48 h) and control (0 h) groups using DESeq2. 7849 and 24,127 differentially expressed genes (DEGs) were obtained from leaves and rhizomes, respectively. GO and KEGG enrichment analyses of the DEGs revealed significantly enriched KEGG pathways, including ribosome, photosynthesis, hormone signal transduction, starch and sucrose metabolism, synthesis of secondary metabolites, and related genes, such as heat shock proteins (HSPs), transcription factors (TFs), and active oxygen scavengers. In conclusion, we conducted the first transcriptome sequencing analysis of the *I. germanica* cultivar ‘Little Dream’ under drought stress and generated a large amount of genetic information. This study lays the foundation for further exploration of the molecular mechanisms underlying the responses of *I. germanica* to drought stress and provides valuable genetic resources for the breeding of drought-resistant plants.

## Introduction

The intensification of the El Niño phenomenon has led to marked changes in global climate patterns. Arid and semiarid areas currently account for approximately 40% of the global land area^1^, but it has been estimated that this percentage will increase to over 50% by the end of the twenty-first century^2^. The damage caused by drought to plants is more serious than that resulting from other abiotic stressors^3^. Due to the rapid development of the global economy, increasing attention has been given to the ecological environment, and substantial developments have been achieved in the construction of gardens; however, with the continuous expansion of urban construction, water consumption and the costs of managing garden green lands have also increased. Therefore, the selection of drought-resistant, water-saving, and arid-environment-tolerant garden plants with excellent ornamental characteristics and identification of the plants’ drought-resistance mechanisms would be of great significance for the cultivation of high-drought-resistant cultivars and the construction of energy-saving gardens.

Plants utilise a complex set of physiological and molecular regulatory mechanisms to resist drought stress. The initial response of plants to drought stress is to prevent water loss by adjusting the opening of stomata^4^. Under continuous drought stress, plants can achieve long-term survival by reducing their photosynthetic and growth rates, hardening their cell walls, and limiting the expansion of their young leaf areas^5^. To protect themselves, plants have evolved an oxidation defence system composed of enzymes and antioxidants that can scavenge reactive oxygen species (ROS) and prevent membrane lipid peroxidation. Osmotic regulatory substances, such as soluble sugars, soluble proteins (SPs), and free proline (Pro), act as osmotic agents or low-molecular-weight molecular chaperones to maintain cell swelling and resist drought stress^6^. The expression profiles of a large number of plant genes are altered in response to drought stress, and these genes can be divided into two main categories according to their functions^7^. The first category involves genes such as those encoding transcription factors (TFs), plant hormones, and phosphatases, which contribute to transcriptional regulation and signalling cascades, and the second category comprises genes encoding HSPs, photosynthesis-related factors, osmotic protective agents, and antioxidant synthesis genes as well as other effector proteins that protect plants from abiotic stress. In general, TFs recognize specific elements in the promoters of stress-resistance genes and act to regulate their transcription. MYB, NAC, WRKY, and other TF families contribute to drought stress regulation in many plants and have been studied in *Arabidopsis thaliana*^8^, barley^9^, rice^10^, and soybean^11^; however, few related studies have been performed in garden plants. Simultaneously, plant metabolic pathways, such as ribosome, photosynthesis, hormone signal transduction, phenylpropane biosynthesis, starch and sucrose metabolism, and terpenoid and flavonoid biosynthesis, are also involved in abiotic stress. The balance among these pathways is important for plant stress resistance.

The development of high-throughput sequencing has prompted research progress in genomics and transcriptomics. Due to its high-throughput format as well as its high sensitivity and accuracy^12^, RNA-seq is an effective transcriptome analysis method that has been widely used to determine the expression profiles and gene structures of model and non-model plants under stress and can thus facilitate the analysis of complex biochemical processes. Furthermore, high-throughput sequencing can be used to identify unknown and rare transcripts and to accurately analyse gene expression differences, gene structure variations, and other genetic characteristics^13^. For non-model plants lacking reference genomes, RNA sequencing promotes research at the molecular level and provides genetic resources for the breeding of resistant cultivars of non-model plants.

*Iris germanica*, which is a common perennial flower used in landscaping, has beautiful leaves, colourful flowers, and numerous cultivars; some of these cultivars have multiple colours and fragrances, and other cultivars flower twice each year. *I. germanica* has the highest ornamental value among the species belonging to the genus *Iris*^14^, and *Iris* species are generally adaptable, resistant to arid conditions, and able to retain water^15^. The drought resistance of *I. germanica* is superior to that of other *Iris* species, which makes *I. germanica* an ideal plant for landscaping. To date, research on the drought resistance of *Iris* species has mainly focused on interspecific comparisons; most of the few studies that have investigated *I. germanica* cultivars have examined only physiological-level changes^16^, and the molecular mechanisms underlying drought resistance thus remain poorly understood.

In this study, by evaluating various physiological indices of 10 *I. germanica* cultivars with excellent drought-stress-resistance characteristics, we selected a cultivar with strong drought resistance, ‘Little Dream,’ for further study. The leaves and rhizomes of ‘Little Dream’ plants treated with 20% PEG-6000 solution for 0, 6, 12, 24, and 48 h were harvested and used to construct cDNA libraries for sequencing on the Illumina platform. The differential gene expression profiles of selected tissues in response to drought for various periods of time were then analysed. The differentially expressed genes (DEGs) were functionally annotated, and the pathways related to the drought tolerance of ‘Little Dream’ were subjected to an in-depth analysis, which enabled the identification of potential drought-resistance candidate genes. This study provides the first transcriptome map of *I. germanica* in response to drought stress and data regarding the changes in the gene expression profiles of different tissues under stress for multiple time points, which will be useful for further analysis of the molecular mechanisms underlying drought resistance. The development of garden plants at the molecular level lags far behind that of crops. The results of our study will promote molecular research on garden plants, enrich plant genetic resources, and lay the foundation for the molecular breeding of drought-resistant *Iris* cultivars.

## Results

### Changes in physiological indices under drought stress

The changes in physiological indices among the tested *I. germanica* cultivars indicated certain differences among the cultivars in terms of their drought-resistance abilities (Supplementary Table S1). With increases in drought stress, the chlorophyll (Chl) content first increased and then decreased generally in all the cultivars. The Chl content in ‘Blood Stone’ increased to approximately 3.87 times that of the control, whereas the smallest increase (to 0.27 times that of the control) was detected in ‘Tantara’. The Chl content of ‘Tantara’ reached a nadir with a value of 0.24 ± 0.10 mg g^−1^ after 30 days of drought stress and decreased by 84.31% compared with that of the control, and this decrease represented the largest decrease detected among the cultivars. The Chl content of ‘Little Dream’ reached a minimum value of 0.72 ± 0.27 mg g^−1^, and this decrease (only 4% less than that of the control) was the smallest among the cultivars.

The relative electric conductivity (REC) of the leaves displayed an increasing trend in the 10 cultivars and reached a peak after 30 days. The smallest increase was observed in ‘Little Dream’ (1.90 times the control), which indicated that the cell membranes showed the least damage in this cultivar and that this cultivar exhibited strong drought resistance. The largest increase was detected in ‘Immortality’ (3.58 times the control), which indicated that this cultivar exhibited the most serious cell membrane damage under drought stress.

The observed changes in SOD activity were complex, and the levels of the changes and time points at which extreme values were detected differed among the 10 cultivars. The SOD activity of ‘Tantara’ was 100.08 ± 15.38 U g^−1^ at 18 days, and this value represented the largest decrease among the cultivars and was 79.73% of the control value. The SOD activity of ‘Memory of Harvest’ was 825.51 ± 152.61 U g^−1^ at 18 days, which was significantly higher than and reached 2.74 times the value at 0 days. The SOD activities of ‘X’ Brassie,’ ‘Music Box,’ and ‘Little Dream’ were highest at 0 days, and declines of 44.17% and 48.76% of the control values were detected at 12 days in ‘Music Box’ and ‘Little Dream,’ respectively, whereas the SOD activity of ‘X’ Brassie’ was lowest after 24 days of stress (42.73% of the control value). In these three cultivars, the SOD activity began to increase significantly from 24 to 30 days, possibly due to the strong adaptability of the cultivars, and no substantial physiological impact was detected at the early stage of drought.

The changes in the MDA content observed in the 10 cultivars under drought stress were not the same. The MDA content first increased, then decreased and then increased again in all of the cultivars with the exception of ‘X’ Brassie,’ ‘Immortality,’ and ‘White and Gold,’ in which the content displayed a trend of first decreasing and then increasing. The MDA content exhibited the highest increase in ‘Immortality,’ where it reached peak of 33.48 ± 1.11 nmol g^−1^ (2.31 times the level at 0 days) at 30 days. In contrast, the MDA content exhibited the lowest increase in ‘Cherry Garden,’ and the peak in this cultivar was 1.36 times the level at 0 days.

The Pro content initially decreased, then increased, and finally declined in all 10 cultivars. The greatest increase was detected in ‘Tantara,’ where it reached a peak value of 868.75 ± 159.22 μg g^−1^ (12.40 times the level at 0 days) at 18 days, whereas the Pro content of ‘Immortality’ reached a peak value of 673.35 ± 112.83 μg g^−1^, which was 1.44 times that at 0 days and represented the smallest increase among all the cultivars.

The SP content initially increased, then decreased, and then fluctuated around a steady state in all 10 cultivars. The greatest increase was found in ‘Memory of Harvest’ (3.32 times that at 0 days), whereas the smallest increase was detected in ‘Clarence’ (2.67 times that at 0 days).

### Comprehensive evaluation of drought resistance

The analysis of membership function showed that the average membership degree of the 10 cultivars ranged from 0.38 to 0.67. ‘Little Dream’ exhibited the highest average membership of 0.67, whereas ‘Clarence’ had the lowest value of 0.38. According to the average membership degree, the 10 cultivars were ordered in terms of drought resistance as follows: ‘Little Dream’ > ‘Music Box’ > ‘X’ Brassie’ > ‘Blood Stone’ > ‘Cherry Garden’ > ‘Memory of Harvest’ > ‘Immortality’ > ‘White and Gold’ > ‘Tantara’ > ‘Clarence’ (Table 1).

**Table 1:** Value for membership function for *I. germanica.*

Cultivars	Chl	REC	SOD	MDA	SP	Pro	Average membership	Rank
Cherry garden	0.00	0.89	0.83	0.25	0.96	0.84	0.63	5
Tantara	0.55	1.00	0.00	0.00	1.00	0.03	0.43	9
Blood stone	0.89	0.20	0.87	0.15	0.93	0.82	0.64	4
Music box	0.58	0.77	0.70	0.16	0.90	0.84	0.66	2
Memory of harvest	0.72	0.01	0.36	1.00	0.37	0.60	0.51	6
Clarence	0.60	0.89	0.22	0.50	0.00	0.08	0.38	10
X’Brassie	1.00	0.18	0.92	0.17	0.69	1.00	0.66	3
Little dream	0.78	0.16	1.00	0.59	0.91	0.58	0.67	1
Immortality	0.27	0.87	0.91	0.27	0.66	0.02	0.50	7
White and gold	0.95	0.00	0.74	0.56	0.62	0.00	0.48	8

Chl, chlorophyll; REC, relative electric conductivity; SOD, superoxide dismutase; MDA, malondialdehyde; SP, soluble protein; Pro, free proline.

### Sequence analysis and read assembly

Libraries of cDNA were constructed from 30 samples of ‘Little Dream’ leaves and rhizomes exposed to 20% PEG-6000 stress for 0, 6, 12, 24, and 48 h and sequenced using the Illumina HiSeq X Ten platform. We acquired approximately 2.163 billion raw reads and filtered more than 2.113 billion clean reads. The numbers of clean reads obtained for the leaves and rhizomes were 1.071 billion and 1.042 billion, respectively. The total number of bases with a phred value > 20 was 96.70%. Trinity splicing generated 1, 976, 033 transcripts with a mean length of 660 bp, and 743, 982 unigenes were obtained by hierarchical clustering of the transcripts using Corset (https://code.google.com/p/corset-project/). The maximum length, minimum length, mean length, median length, N50, and total nucleotide number of the unigenes were 14, 851 bp, 201 bp, 716 bp, 479 bp, 910 bp, and 532, 972, 188 nt, respectively, and the mean guanine-cytosine (GC) content was 50.45% (Table 2). Most unigenes (282, 502) were 301–500 bp in length, and these accounted for 37.97% of the total unigenes; 213, 658 unigenes (28.72%) were 501–1000 bp (14.81%), 110,158 unigenes were less than 301 bp, 99, 745 unigenes (13.41%) were 1001–2000 bp, and 37,919 unigenes (5.10%) were more than 2000 bp in length (Supplementary Fig. S1). The Illumina reads generated in this study have been deposited with the National Center for Biotechnology Information (NCBI) and are available in the Short Read Archive (SRA) Sequence Database under the accession number SRP278149.

**Table 2:** Overview of the sequencing and assembly.

Items	Number
Unigenes	743,982
Maximum length (bp)	14,851
Minimum length (bp)	201
Mean length (bp)	716
Median length (bp)	479
N50 (bp)	910
Total nucleotide number(nt)	532,972,188
GC percentage (%)	50.45%

### Functional annotation and classification of unigenes

**Figure 1:**
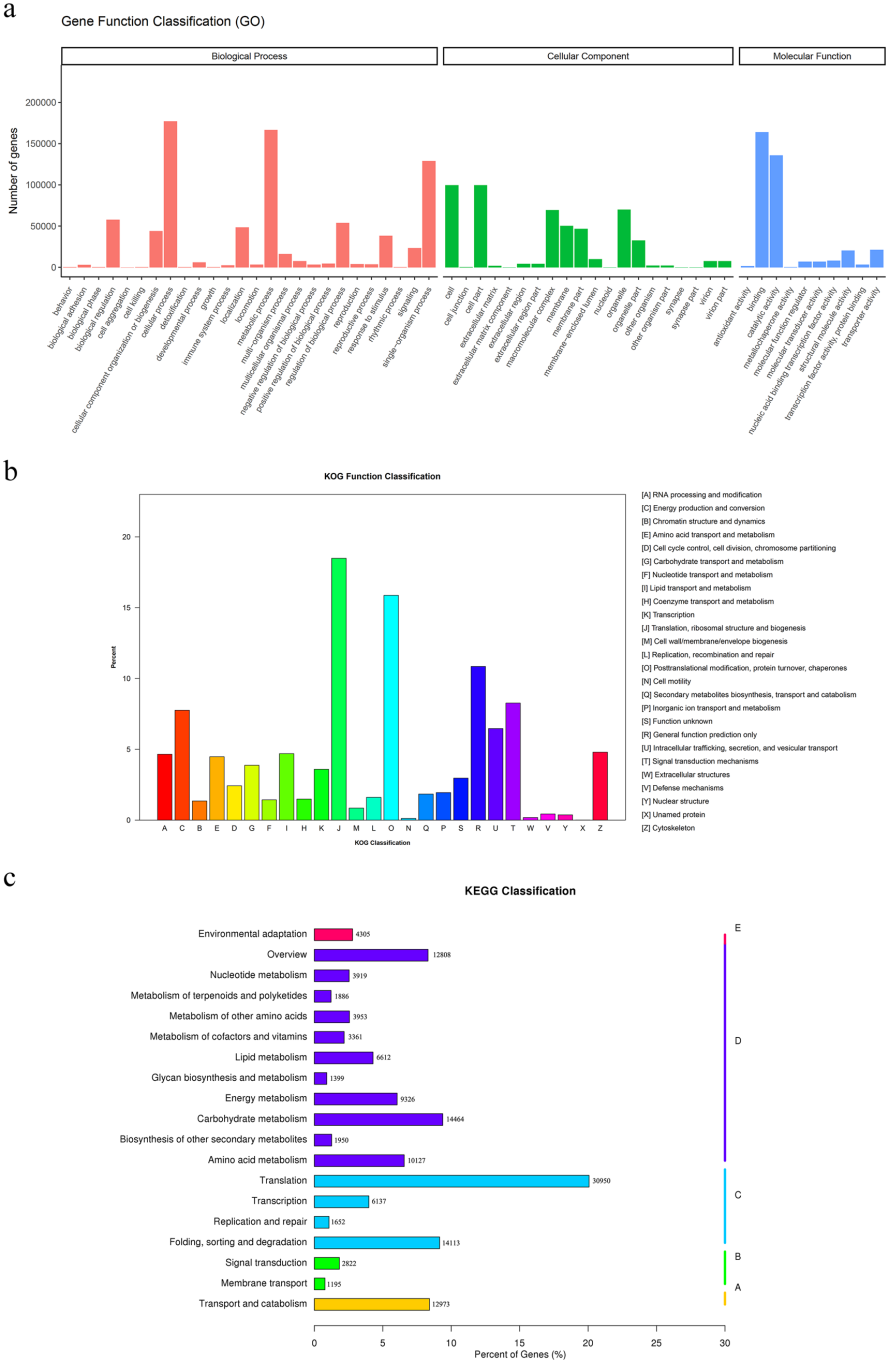
Gene ontology classification of assembled unigenes (**a**). KOG function classification of assembled unigenes (**b**). Functional classification and pathway assembled unigenes by KEGG (**c**); The A–E indicates Cellular Processes, Environmental Information Processing, Genetic Information Processing, Metabolism, Organismal Systems, respectively.

To obtain comprehensive gene function information, all the unigenes were compared with seven databases, and the numbers of unigenes successfully annotated using the Nr, Nt, KO, Swiss-Prot, Pfam, GO, and KOG databases were 253, 193 (34.03%), 125, 324 (16.84%), 154, 223 (20.72%), 270, 450 (36.35%), 319, 221 (42.90%), 319, 221 (42.90%), and 168, 759 (22.68%), respectively (Table 3). Overall, 432, 431 (58.12%) unigenes were successfully annotated in at least one database, whereas no functional annotation was obtained for 311, 551 (48.88%), which was likely due to matching with proteins of unknown function or an absence of similar nucleotide sequences. These data provide new transcript information. According to the comparison with the Nr database, the top five annotated species were *Quercus suber* (16.1%), *Asparagus officinalis* (7.6%), *Elaeis guineensis* (2.7%), *Phoenix dactylifera* (2.4%), and *Guillardia theta* (2.4%) (Supplementary Fig. S2). GO is a classification system that comprehensively describes the attributes of genes and their products in organisms. This system has three aspects, and these are used to describe the biological process, cellular component, and molecular function features of genes. Within the biological process domain, the assignments were mostly enriched in the terms cell process (177,023, 55.45%), metabolic process (166,627, 52.20%), and single-organism process (128,928, 40.39%), and in the cellular component domain, cell and cell part (99,633, 31.21%), macromolecular complex (69,437, 21.75%), and organelle part (32,512, 10.18%) were the most strongly enriched terms. In the molecular function domain, the clearest matches were to the terms binding (163,755, 51.30%) and catalytic activity (135,871, 42.56%) (Fig. 1a). According to the KOG annotation, the successfully annotated unigenes were classified based on the 26 KOG groups, and among these, 31,199 (18.49%) were classified as translation, ribosomal structure, and biogenesis, which accounted for the highest proportion, followed by posttranslational modification, protein turnover, and chaperones (26,778, 15.87%) (Fig. 1b). After the KO annotations of the unigenes, the biochemical pathways regulated in the leaves and rhizomes of ‘Little Dream’ were identified by KEGG metabolic pathway analysis. A total of 154,223 unigenes were annotated and divided into 131 KEGG pathways (Supplementary Table S2). Following the removal of unigenes related to human disease, the annotated unigenes were divided into five branches: cellular processes, environmental information processing, genetic information processing, metabolism, and organismal systems. Translation (30,950, 20.06%); folding, sorting, and degradation (14,113, 9.15%) in the genetic information processing branch; and carbohydrate metabolism (14,464, 9.37%) in the metabolism branch were the three most represented metabolic pathways (Fig. 1c).

**Table 3:** Statistics of unigenes annotation success rate.

Annotated database	Number of genes	Percentage (%)
Annotated in Nr	253,193	34.03
Annotated in Nt	125,324	16.84
Annotated in KO	154,223	20.72
Annotated in Swiss-Prot	270,450	36.35
Annotated in Pfam	319,221	42.9
Annotated in GO	319,221	42.9
Annotated in KOG	168,759	22.68
Annotated in all databases	38,058	5.11
Annotated in at least one database	432,431	58.12
Total unigenes	743,982	100

### Analysis of the DEGs in different tissues

**Figure 2:**
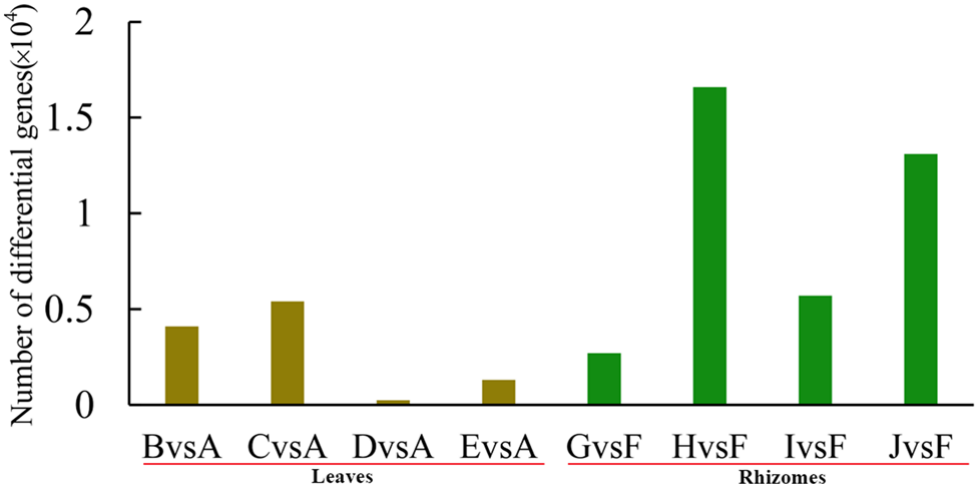
The number of DEGs from samples of leaf and rhizome at each treatment time point compared with the control (The A-E indicates the ‘Little Dream’ of leaves under stress at 0, 6, 12, 24, and 48 h, respectively, the F-J were ‘Little Dream’ of rhizomes under stress at the same time points).

**Figure 3:**
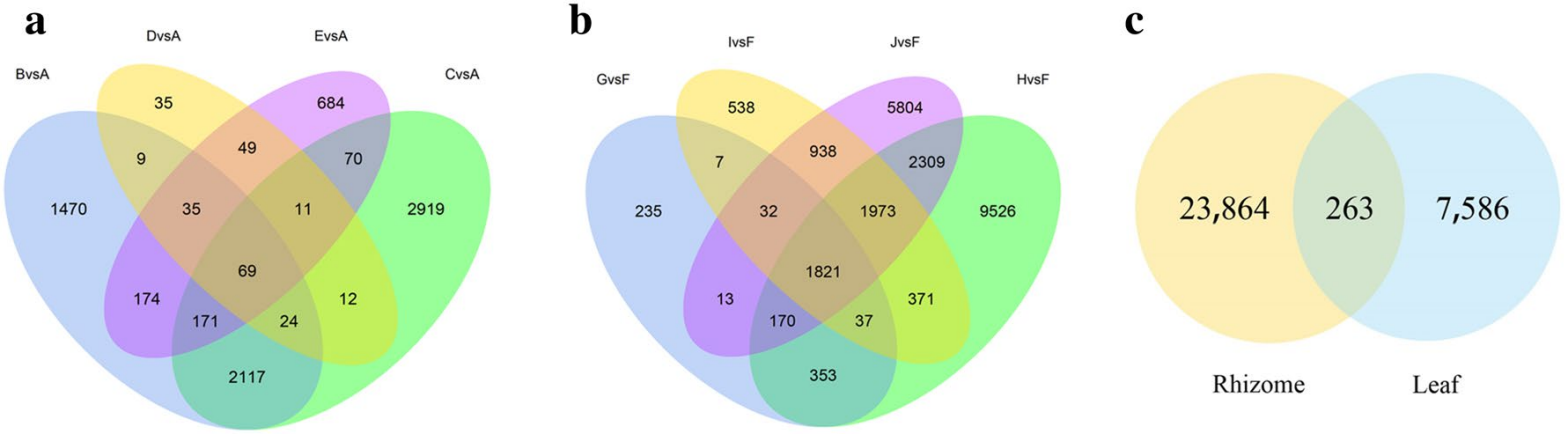
The Venn diagram shows the number of DEGs in samples of leaf (**a**); rhizome (**b**); leaf and rhizome (**c**) (The A-E indicates the ‘Little Dream’ of leaves under stress at 0, 6, 12, 24, and 48 h, respectively, the F-J were ‘Little Dream’ of rhizomes under stress at the same time points).

The read count data obtained from the analysis of gene expression levels were analysed using DESeq2^17^. The filtering thresholds were padj < 0.05 and |log2 Fold-Change|> 1. Among those that met these thresholds, the DEGs that met the criterion log2 Fold-Change > 0 were considered upregulated, and the others were considered downregulated. We counted the number of DEGs at four time points during treatment with 20% PEG-6000 stress (Fig. 2). Using 0 h as the control reference value, the number of DEGs in leaves subjected to drought stress for 6, 12, 24, and 48 h was 4069 (2516 upregulated and 1553 downregulated), 5393 (3238 upregulated and 2155 downregulated), 244 (211 upregulated and 33 downregulated), and 1263 (987 upregulated and 276 downregulated), respectively. A total of 7849 DEGs were found, and these included 69 (65 upregulated and 4 downregulated) DEGs that were found at all four time points (Fig. 3a). The number of DEGs in the rhizome exposed to drought stress for 6, 12, 24, and 48 h was 2668 (172 upregulated and 2496 downregulated), 16,560 (1477 upregulated and 15,063 downregulated), 5717 (17 upregulated and 5700 downregulated), and 13,060 (100 upregulated and 12,960 downregulated), respectively. A total of 24,127 DEGs were identified, and these included 1821 (all downregulated) that were found at all four time points (Fig. 3b). Furthermore, 263 DEGs were found in both the leaves and rhizomes (Fig. 3c).

**Figure 4:**
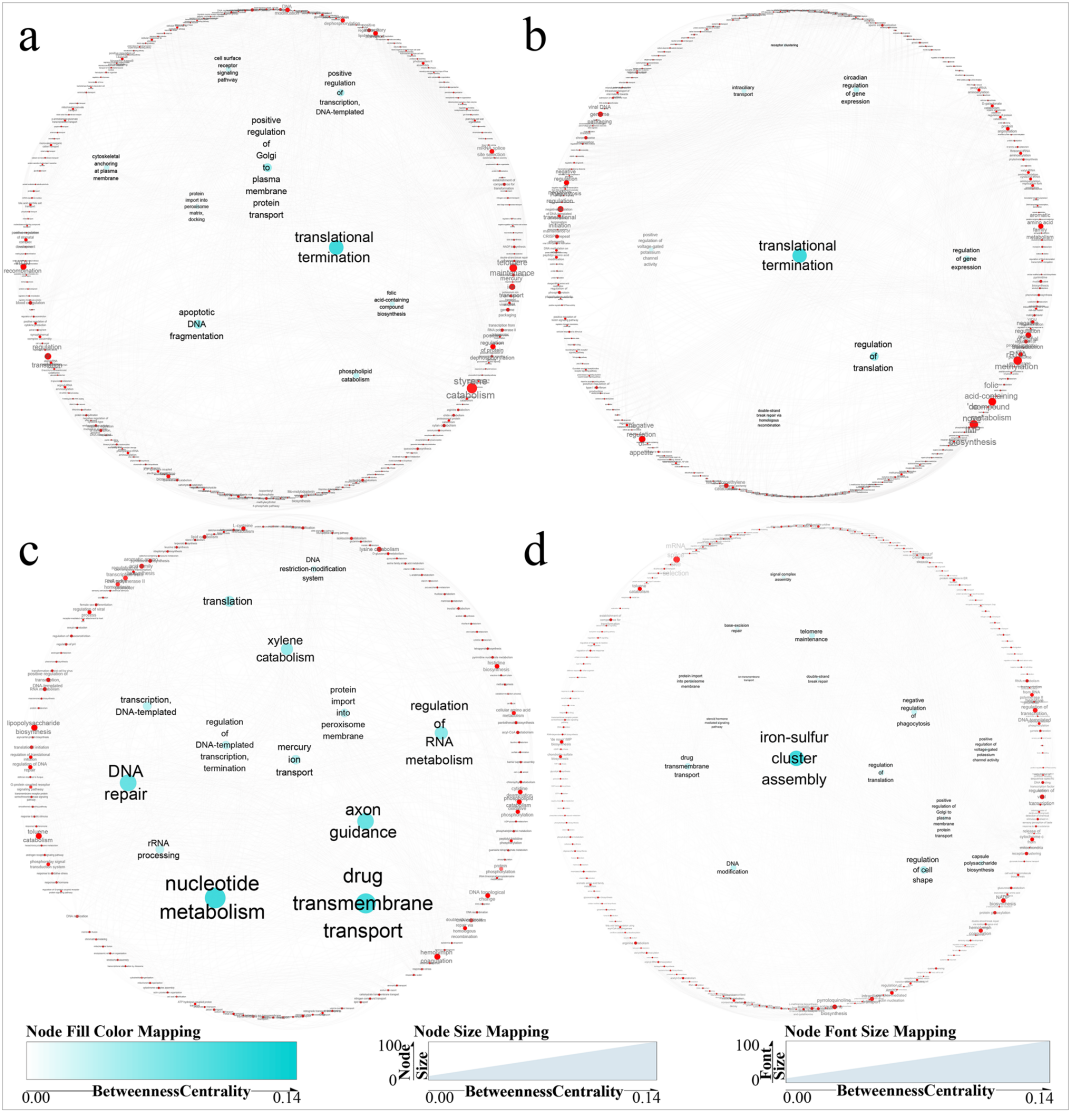
GO function annotation of short-acting genes in leaf (**a**); in rhizome (**b**). Go function annotation of long-acting genes in leaf (**c**); in rhizome (**d**).

A statistical analysis of the number of DEGs revealed an interesting phenomenon. In both the leaves and rhizomes, the number of DEGs reached a peak at 12 h and then decreased sharply before beginning to increase again at 48 h. We speculated that this phenomenon might be related to the different response times of genes to drought stress. Therefore, we named the DEGs detected at 6 and 12 h short-acting genes because these exhibited instant/rapid changes in expression, whereas the DEGs detected at 24 and 48 h were defined as long-acting genes because these exhibited slow changes in expression. Some of these genes were expressed at both the early and late stages of exposure to stress, which indicated that they play roles in the primary and later tolerance responses to stress. The short- and long-acting genes in the different tissues were arranged according to their GO functional annotation. The files were imported into the REVIGO web server (http://revigo.irb.hr/) and processed using Cytoscape software (version 3.8.2)^18^ to generate Fig. 4. The results indicated that long- and short-acting genes had different functions in leaves and rhizomes. In leaves, short-acting genes were mainly active in the processes of gene expression, protein synthesis, and transportation (Fig. 4a), whereas the functions of short-acting genes in rhizomes were primarily enriched in transcription and expression (Fig. 4b). The functions of long-acting genes in leaves were focused on DNA repair, transmembrane transport of substances, and the regulation of RNA synthesis (Fig. 4c), whereas those in rhizomes were mainly related to ferritin and transmembrane ion transport (Fig. 4d).

### Functional classification and cluster analysis of DEGs

**Figure 5:**
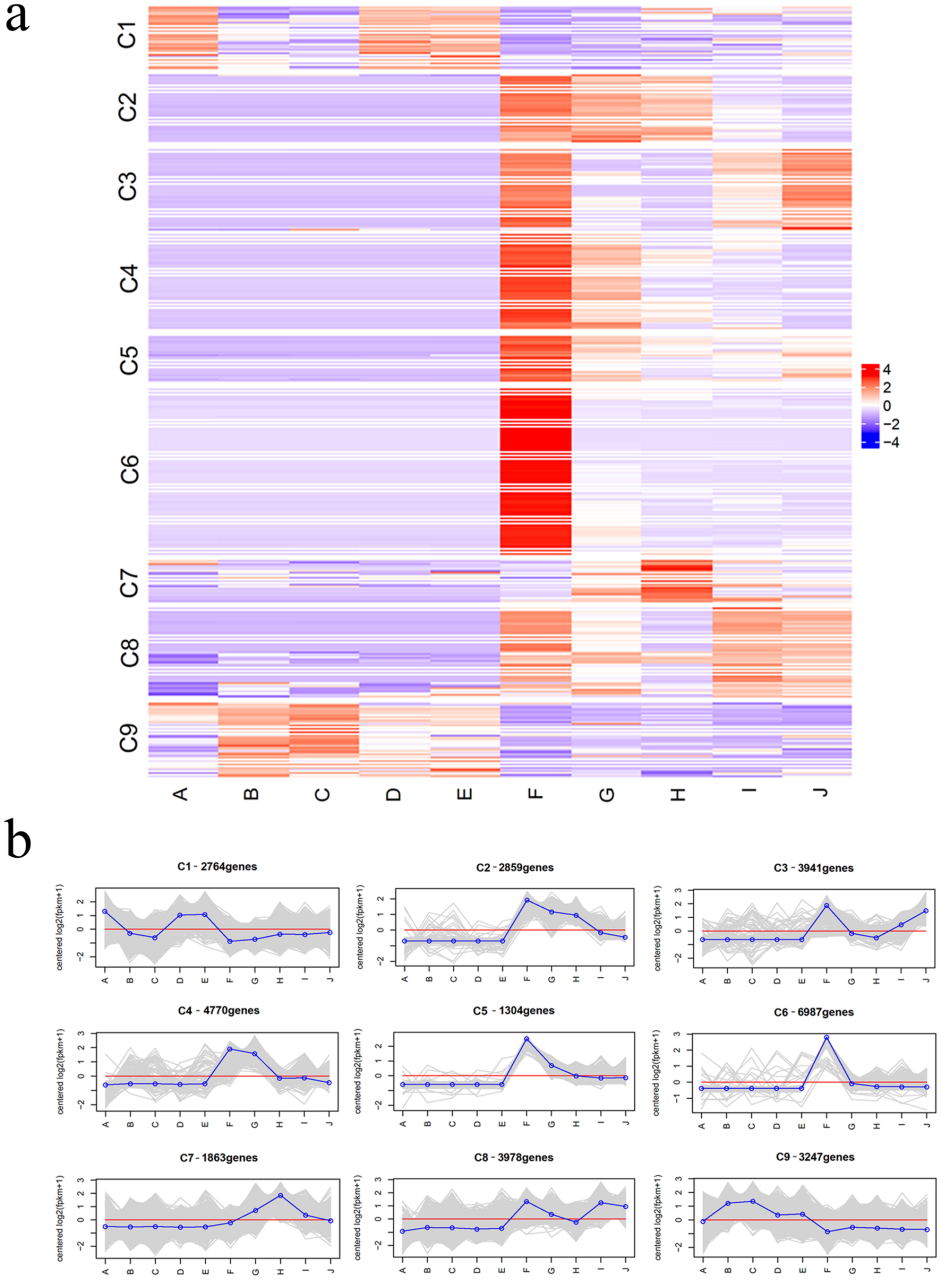
Gene expression patterns across leaf and rhizome samples. Heatmap of 31, 713 DEGs that were grouped into nine clusters by k-mean method (**a**); The average value of log-transformed FPKM value for genes in cluster (**b**).

Based on K-means clustering and a Pearson correlation analysis, the expression patterns and cluster analysis of the 31,713 DEGs were determined using R software (https://cran.r-project.org/sources.html) with the Complex Heatmap (https://github.com/jokergoo/ComplexHeatmap)^19^ and circlize packages (https://github.com/jokergoo/circlize)^20^. The number of clusters was set to nine, and the 31,713 DEGs were then divided into nine clusters according to their expression patterns (Fig. 5a,b). We also conducted biological function analyses of each cluster (Supplementary Table S3).

As shown in Fig. 5, clusters C2 to C8 included genes expressed at low levels in leaves but at high levels in rhizomes, and differences were found among these clusters. In rhizomes, the expression of genes in the C2 cluster was significantly induced from 0 to 12 h and then decreased after 24 h. These genes were enriched in organonitrogen compound biosynthetic processes and ribosomes. The expression of the genes in C3 was markedly induced at 0 h, decreased from 6 to 12 h, and then gradually increased from 24 to 48 h. These genes were functionally enriched in oxidation–reduction processes, oxidoreductase activity, haem binding, tetrapyrrole binding, glucose metabolism, and glutamate biosynthesis. The genes in the C4 and C5 clusters were highly expressed from 0 to 6 h and then showed decreases in expression; however, the expression of the genes in the C5 cluster at 48 h was higher than that of the genes in the C4 cluster. The genes in the C4 cluster were abundantly enriched in carbohydrate catabolism, thylakoid-related tissues, glucose metabolism, photosynthesis, nucleoside diphosphate metabolism, and glycolysis, whereas those in the C5 cluster were enriched in hydrolase activity, carbohydrate metabolism, TF activity, and sequence-specific DNA binding, among other functions. The expression of the genes in the C7 cluster was low at 0 h, slowly increased from 6 to 12 h, reached a peak at 12 h, and then gradually decreased. These genes were most enriched in biosynthesis, organic nitrogen synthesis and metabolism, catalytic activity, oxidoreductase activity, and metabolism functions. The expression of the genes in the C8 cluster was high from 0 to 6 h, then declined slowly to reach a nadir at 12 h, and then increased from 24 to 48 h. This gene cluster was functionally enriched in ribosome-related pathways, translation, peptide biosynthesis metabolism, protein metabolism, gene expression, and primary metabolism. Compared with those in the other clusters, the genes in the C6 cluster showed their highest expression at 0 h and subsequently remained at low levels in the rhizome, and the functional enrichment of this cluster was very similar to that of the C8 cluster.

The C1 and C9 clusters included genes expressed at high and low levels in leaves and rhizomes, respectively. In leaves, the expression of the genes in the C1 cluster was high at 0 h, low from 6 to 12 h, and then significantly increased from 24 to 48 h. Their functional enrichment analysis suggested their involvement in DNA integration. The expression of the genes in the C9 cluster was relatively low at 0 h and stably high from 6 to 48 h. These genes were enriched in cysteine peptidase activity, protein metabolism, amide biosynthesis metabolism, translation, and calcium binding.

The genes in clusters C2 to C8 were almost exclusively expressed in the rhizome, whereas most genes in the C1 and C9 clusters were only expressed in leaves, which revealed that these genes exhibit clear tissue specificity. The genes expressed in rhizomes were markedly more abundant than those expressed in leaves. An analysis of the DEGs in clusters C1 to C9 demonstrated that the plant’s biological regulation in response to PEG-6000 treatment was dependent on dynamic gene coordination.

### GO and KEGG analyses of DEGs

**Figure 6:**
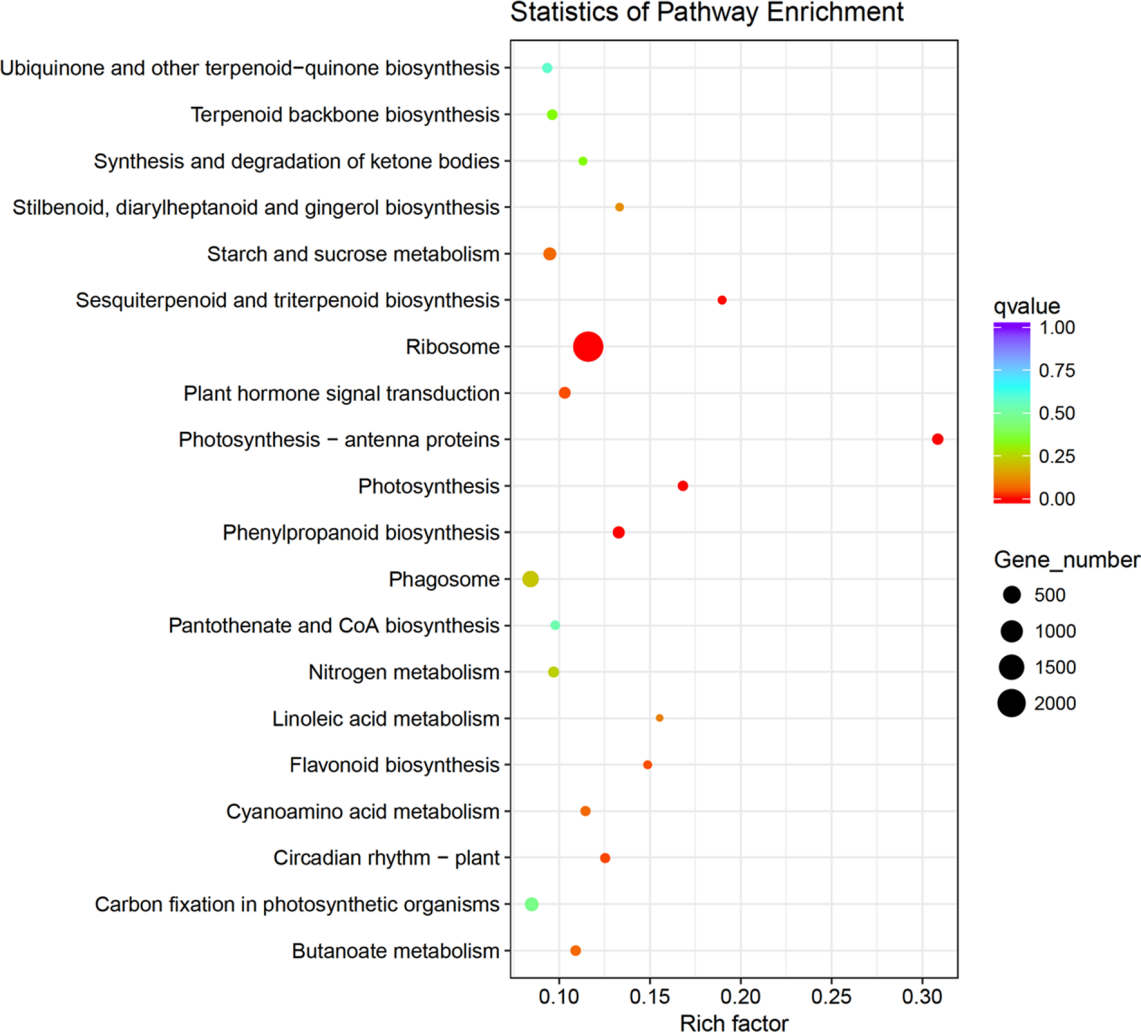
KEGG pathway enrichment scatter diagram of DEGs. Only the top20 most strongly represented pathways are displayed in the diagram. The degree of KEGG pathway enrichment is represented by an enrichment factor, the q-value, and the number of unigenes enriched in a KEGG pathway. The enrichment factor indicates the ratio of differential expression unigenes enriched in this pathway to the total number of annotated unigenes in this pathway. The names of the KEGG pathways are listed along the y-axis. The q-value indicates the corrected p-value, ranging from 0–1, and qvalue closer to 0 indicates greater enrichment.

We then conducted GO and KEGG pathway analyses of the DEGs. A total of 4881 GO terms were assigned to the 31,713 DEGs that responded to PEG-6000 treatment, and 1341 of these GO terms were enriched in nine clusters (Supplementary Fig. S3a). In the biological process category, metabolic, cellular, and organic substance metabolism processes were prominently represented, and in the cellular component category, cell part and cell were most frequently enriched. In the molecular function category, many genes were assigned to the terms binding, catalytic activity, and organic cyclic compound binding. The KEGG pathway enrichment analysis assigned 11,783 DEGs to 123 KEGG pathways, and 57 of these pathways were enriched in the nine clusters (Supplementary Fig. S3b). These genes were mainly involved in ribosomes, protein processing in the endoplasmic reticulum, phagosomes, and RNA transport, among other functions. The top 20 KEGG pathways are presented in Fig. 6, and the results revealed that ribosome, photosynthesis-antenna proteins, phenylpropanoid biosynthesis, photosynthesis, sesquiterpenoid and triterpenoid biosynthesis, circadian rhythm-plant, flavonoid biosynthesis and plant hormone signal transduction were significantly enriched.

### Response of HSPs to drought stress

The KEGG pathway analysis indicated that the number of genes enriched in protein processing in the endoplasmic reticulum was second only to that enriched in the ribosome: 206 (61 HSP90, 122 HSP70, 21 HSP20, and two other HSP genes) transcripts encoding HSPs were enriched in this pathway. In our study, 18 genes encoding HSP20 were upregulated, two encoding HSP20 were downregulated, and one encoding HSP20 was downregulated at 12 h and upregulated at 48 h in leaves and upregulated at 12 h in rhizomes. Among the upregulated genes, nine transcripts (Cluster-81886.0, Cluster-24469.0, Cluster-34938.262513, Cluster-101316.0, Cluster-34938.24034, Cluster-15367.0, Cluster-15367.0, Cluster-46891.0, Cluster-14520.0, Cluster-34938.18441) met the criterion |log2 Fold-Change| > 5 (Table 4, Supplementary Table S4a).

**Table 4:** List of DEGs of *I. germanica* in response to drought.

Trait name	Sum	Leaf								Rhizome							
		BvsA		CvsA		DvsA		EvsA		GvsF		HvsF		IvsF		JvsF	
		Up-regulated	Down-regulated	Up-regulated	Down-regulated	Up-regulated	Down-regulated	Up-regulated	Down-regulated	Up-regulated	Down-regulated	Up-regulated	Down-regulated	Up-regulated	Down-regulated	Up-regulated	Down-regulated
**Heat shock proteins**																	
HSP90	61	0	0	0	7	0	0	4	0	0	5	0	24	0	12	0	42
HSP70	122	6	1	0	2	0	0	11	0	0	13	7	43	0	21	0	81
HSP20	21	5	0	2	1	0	0	2	0	2	0	13	1	0	0	0	1
Other HSP	2	0	0	0	0	0	0	0	0	0	0	0	0	0	0	0	2
**DEGs related to ROS system**																	
SOD1	8	0	0	0	0	0	0	0	0	0	1	0	4	0	2	0	5
SOD2	15	0	0	0	0	0	0	0	0	0	4	0	8	0	7	0	14
CAT	10	1	0	2	0	0	0	0	1	0	1	0	4	1	3	0	6
POD	16	7	1	4	0	1	1	1	0	0	0	0	1	0	1	0	5
GST	26	2	6	4	3	0	0	0	1	0	1	2	3	0	3	0	9
APX	3	1	0	0	0	0	0	2	0	0	0	0	0	0	0	0	0
AOX	21	1	1	0	1	0	0	0	0	0	0	12	1	0	2	0	6
**DEGs related to phytohormone pathway**																	
Abscisic acid																	
NCED	3	1	0	1	0	2	0	2	0	0	0	0	0	0	0	0	0
PP2C	9	3	0	5	0	5	0	8	0	0	0	0	0	0	0	0	0
PYR/PYL	10	0	8	2	4	0	0	0	4	0	0	0	0	0	0	0	0
SnRK2	8	4	0	6	1	0	0	2	0	0	0	0	0	0	0	0	0
Ethylene																	
ERF2	1	0	0	0	1	0	0	0	0	0	0	0	0	0	0	0	0
AP2	7	4	0	5	0	0	0	0	0	0	0	0	0	0	0	0	0
Auxin																	
IAA	5	3	0	1	0	0	0	1	0	0	0	0	0	0	0	0	0
SAUR	5	2	1	3	1	0	0	1	1	0	0	2	0	0	0	0	0
GH3	3	0	1	0	2	0	0	1	0	0	0	0	0	0	0	0	0
Gibberellic acid																	
DELLA	3	3	0	2	0	0	0	0	0	0	0	0	0	0	0	0	0
Jasmonic acid																	
JAR1	1	0	1	0	1	0	0	0	1	0	0	0	0	0	0	0	0
MYC2	4	1	0	3	0	0	0	0	0	0	0	0	0	0	0	0	0
Brassinsteroid																	
BIN2	2	0	0	0	2	0	0	0	0	0	0	0	0	0	0	0	0
BSK	2	0	1	1	0	0	0	0	0	0	0	0	0	0	0	0	0
**DEGs related to osmotic adjustment substances**																	
Proline metabolism pathway																	
P5CS	3	0	0	0	0	0	0	2	0	0	0	0	1	0	0	0	0
P5CDH	7	0	0	0	0	0	0	0	0	0	1	0	3	0	2	0	5
P4H	3	0	0	0	0	0	0	0	0	0	0	0	3	0	2	0	3
PIP	3	0	1	0	0	0	0	0	0	0	2	0	2	0	1	0	1
PRODH	3	0	0	0	1	0	0	0	0	0	0	0	1	0	0	0	2
G5K	1	0	0	0	0	0	0	0	0	0	0	0	1	0	0	0	0
Starch and sucrose metabolism pathway																	
BGL	23	10	0	5	1	0	0	7	1	0	2	0	6	0	1	0	3
BFF	18	3	1	1	1	1	0	13	1	0	0	0	2	0	0	0	0
SPS	5	4	0	4	1	0	0	0	0	0	0	0	0	0	0	0	0
SS	7	0	0	0	1	0	0	3	1	0	0	2	0	0	0	0	0
glgC	2	1	0	1	0	0	0	1	0	0	0	0	0	0	0	0	0
TPP	3	3	0	3	0	0	0	0	0	0	0	0	0	0	0	0	0
GalAT	3	1	0	2	0	0	0	0	0	0	0	0	0	0	0	0	0
PE	13	3	0	2	0	0	0	6	0	0	0	0	4	0	0	0	0
UGA4E	5	3	0	5	0	0	0	0	0	0	0	0	0	0	0	0	0
UXS1	4	0	0	0	0	0	0	0	0	0	0	1	2	0	1	0	2
TPS	6	0	3	0	3	0	0	0	2	0	0	0	2	0	0	0	0
**DEGs related to biosynthesis of secondary metabolites**																	
Terpenoids																	
PO	2	1	0	0	0	0	0	1	1	0	0	0	0	0	0	0	0
GERD	9	2	0	9	0	0	0	0	0	0	0	0	0	0	0	0	0
HVS	2	1	0	2	0	0	0	0	0	0	0	0	0	0	0	0	0
SM	6	1	0	3	0	1	0	3	0	0	0	1	1	0	0	0	1
Flavonoids																	
CHS	5	1	0	3	0	1	0	1	0	0	2	0	2	0	2	0	2
CA4H	6	6	0	3	0	1	0	4	0	0	0	0	0	0	0	0	0
DFR	1	1	0	0	0	0	0	1	0	0	0	0	0	0	0	0	0
F3H	1	1	0	1	0	0	0	0	0	0	0	0	0	0	0	0	0
F3ʹH	2	2	0	0	0	1	0	1	0	0	0	0	0	0	0	0	0
HCT	6	3	0	2	0	1	0	5	0	0	0	0	0	0	0	0	0
FLS	1	0	0	1	0	0	0	0	0	0	0	0	0	0	0	0	0
Lignin																	
4CL	10	3	0	3	1	0	0	2	0	0	0	0	5	0	0	0	0
CA4H	6	6	0	3	0	1	0	4	0	0	0	0	0	0	0	0	0
F5H	1	1	0	1	0	0	0	0	0	0	0	0	0	0	0	0	0
CALDH	17	3	2	2	1	0	0	2	0	0	3	0	8	0	5	0	7
POD	10	7	1	4	0	1	0	1	0	0	0	0	0	0	1	0	0
COMT	6	3	0	4	0	0	0	0	0	0	0	0	0	0	0	0	0
HCT	6	3	0	2	0	1	0	5	0	0	0	0	0	0	0	0	0
Phenols																	
PAL	5	0	0	5	0	1	0	2	0	0	0	0	0	0	0	0	0

SOD1, superoxide dismutase, Cu–Zn family; SOD2, superoxide dismutase, Fe–Mn family; CAT, Catalase; POD, peroxidase; GST, glutathione S-transferase; APX, ascorbate peroxidase; AOX, alternative oxidase; NCED,9-cis-epoxycarotenoid dioxygenase; PP2C, protein phosphatase 2C; PYL, abscisic acid receptor PYR/PYL family; SnRK2, serine/threonine-protein kinase SRK2; ERF2, ethylene-responsive transcription factor 2; SAUR, Small auxin-up RNA; JAR1, jasmonic acid-amino synthetase; BIN2, protein brassinosteroid insensitive 2; BSK, BR-signaling kinase; P5CS, delta-1-pyrroline-5-carboxylate synthetase; P5CDH, 1-pyrroline-5-carboxylate dehydrogenase; P4H, prolyl 4-hydroxylase; PIP, proline iminopeptidase; PRODH, proline dehydrogenase; G5K, glutamate 5-kinase; BGL, beta-glucosidase; BFF, beta-fructofuranosidase; SPS, sucrose-phosphate synthase; SS, sucrose synthase; glgC, glucose-1-phosphate adenylyl transferase; TPP, trehalose 6-phosphate phosphatase; GalAT, α-1,4-galacturonyl transferase; PE, pectinesterase; UGA4E, UDP-glucuronide 4-episomerase; UXS1, UDP-glucuronate decarboxylase; TPS, trehalose 6-phosphate synthase/phosphatase; PO, premnaspirodiene oxygenase; GERD, (-) -germacrene D synthase; HVS, vetispiradiene synthase; SM, squalene monooxygenase; CHS, chalcone synthase; CA4H, trans-cinnamate 4-monooxygenase; DFR, bifunctional dihydroflavonal 4-reductase/flavanone 4-reductase; F3H, naringenin 3-dioxygenase; F3ʹH, flavonoid 3ʹ-monooxygenase; HCT, shikimate O-hydroxy cinnamoyl transferase; FLS, flavonol synthase; 4CL, 4-coumarate–CoA ligase; F5H, ferulate-5-hydroxylase; CALDH, cinnamyl-alcohol dehydrogenase; COMT, caffeic acid 3-O-methyltransferase; PAL, phenylalanine ammonia-lyase.

### Response of antioxidants to drought stress

The transcriptomic study identified 23 SOD-related genes (eight SOD1 and 15 SOD2), all of which were downregulated in rhizomes. Ten catalase (CAT)-related genes were detected: two were upregulated, seven were downregulated, and one was upregulated at 12 h and downregulated at 48 h in leaves. Sixteen peroxidase (POD)-related genes were identified, and these included eight upregulated and eight downregulated genes (six glutathione peroxidase genes were downregulated). Twenty-six glutathione S-transferase (GST)-related genes, including five upregulated and 21 downregulated genes, were found. In addition, three l-ascorbate peroxidase genes were upregulated. Furthermore, 21 alternative oxidase (AOX)-related genes were detected, and 13 of these were upregulated (Table 4, Supplementary Table S4b).

### Response of the plant hormone signal transduction pathway to drought stress

In this study, several DEGs related to abscisic acid (ABA), ethylene (ET), auxin (AUX), gibberellic acid (GA), jasmonic acid (JA) and brassinosteroid (BR) signal transduction were found. In the ABA signalling pathway, we identified three genes related to 9-cis-epoxycarotenoid dioxygenase (NCED), and all of these were upregulated under drought stress. Nine protein phosphatase 2C (PP2C) genes were found, and all of these genes were upregulated in leaves. Ten PYR/PYL genes, including one upregulated, eight downregulated, and one upregulated at 12 h and downregulated at 48 h, were detected. Eight SnRK2 genes, including seven upregulated and one downregulated, were also identified. Under ET signalling, one ERF2 gene was downregulated and seven AP2 genes were upregulated in leaves. In AUX hormonal signalling, all five auxin-reactive protein IAA-related genes were upregulated. The expression of four small auxin-up RNA (SAUR) genes was upregulated, whereas one SAUR gene was downregulated at 6, 12, and 48 h in leaves and upregulated at 12 h in rhizomes, and three GH3 family genes, including one upregulated and two downregulated genes, were identified. Three DELLA proteins were upregulated in GA under drought stress. In the JA pathway, one jasmonic acid-amino synthetase (JAR1) was downregulated, whereas four MYC2 family genes were upregulated. In BRs, two protein brassinosteroid insensitive 2 (BIN2) proteins, both of which were downregulated at 12 h in leaves under stress, were identified, and two BR-signalling kinases (BSKs), one of which was upregulated at 12 h and the other of which was downregulated at 6 h in leaves under drought stress, were identified (Table 4, Supplementary Table S4c).

### Response of osmotic adjustment substance-related genes to drought stress

In the proline metabolic pathway, we identified three delta-1-pyrroline-5-carboxylate synthetase (P5CS)-related genes, including two upregulated and one downregulated genes. In addition, seven 1-pyrroline-5-carboxylate dehydrogenase (P5CDH), three prolyl 4-hydroxylase (P4H), three proline iminopeptidase (PIP), three proline dehydrogenase (PRODH), and one glutamate 5-kinase (G5K) genes were downregulated. In the starch and sucrose metabolism pathway, some genes encoding beta-glucosidase (BGL), beta-fructofuranosidase (BFF), sucrose-phosphate synthase (SPS), sucrose synthase (SS), glucose-1-phosphate adenylyl transferase (glgC), trehalose 6-phosphate phosphatase (TPP), α-1,4-galacturonyl transferase (GalAT), pectinesterase (PE), UDP-glucuronide 4-episomerase (UGA4E), and UDP-glucuronate decarboxylase (UXS1) were upregulated, and six genes encoding trehalose 6-phosphate synthase/phosphatase (TPS) were downregulated (Table 4, Supplementary Table S4d).

### Response of secondary metabolite biosynthesis-related genes to drought stress

In this study, sesquiterpenoid, triterpenoid, flavonoid, and phenylpropanoid biosynthesis was significantly enriched according to the KEGG pathway analysis. Twenty-six genes related to sesquiterpenoid and triterpenoid biosynthesis were identified, and 17 of these genes, including one premnaspirodiene oxygenase (PO) gene, nine (–)-germacrene D synthase (GERD) genes, two vetispiradiene synthase (HVS) genes, and five squalene monooxygenase (SM) genes, were upregulated. In the flavonoid biosynthesis pathway, 25 genes related to flavonoid biosynthesis were found, and among these, 20, including three chalcone synthase (CHS) genes, six trans-cinnamate 4-monooxygenase (CA4H) genes, one bifunctional dihydroflavanol 4-reductase/flavanone 4-reductase (DFR) gene, one naringenin 3-dioxygenase (F3H) gene, two flavonoid 3ʹ-monooxygenase (F3ʹH) genes, six shikimate O-hydroxy cinnamoyl transferase (HCT) genes, and one flavonol synthase (FLS) gene, were upregulated. Seventy enzymes related to lignin formation were detected in the phenylpropane biosynthesis pathway, and 34 of these genes, including four 4-coumarate-CoA ligase (4CL) genes, six CA4H genes, one ferulate-5-hydroxylase (F5H) gene, three cinnamyl-alcohol dehydrogenase (CALDH) genes, eight POD genes, six caffeic acid 3-O-methyltransferase (COMT) genes, and six HCT genes, were upregulated. Furthermore, five phenylalanine ammonia-lyase (PAL) genes related to phenolic synthesis were identified and upregulated (Table 4, Supplementary Table S4e–h).

### Response of transcription factor families to drought stress

A total of 82 TF families were identified, 11,099 TF genes were expressed in at least one sample, and 1047 TF genes were classified into 70 families that showed significantly different responses to PEG-6000 stress. The three most abundant TF families were Zn-clus (124), C2H2 (90), and C3H (78), followed by Orphans (70), MYB (68), CCAAT (58), HMG (45), bHLH (37), bZIP (29), FHA (25), GNAT (25), HSF (24), NAC (23), AP2-EREBP (22), SET (21), HB (21), WRKY (20), and CSD (20). We inspected the expression levels of Zn-clus, C2H2, C3H, MYB, bHLH, bZIP, HSF, NAC, and WRKY TFs and observed different expression patterns (Supplementary Fig. S4). The genes belonging to the MYB, bHLH, WRKY, and NAC families exhibited diverse expression patterns in different tissues and across treatment times, and the members of these families showed different responses to PEG stress. In contrast, most genes belonging to the Zn-clus, C2H2, C3H, bZIP, and HSF families were expressed at low and high levels in leaves and rhizomes, respectively, and their expression peaked at 0 h in rhizomes under PEG stress. In rhizomes, the levels of Zn-clus family genes decreased gradually after 6 h, reached a nadir at 12 h, and then increased from 24 to 48 h. The expression of most genes belonging to the C2H2 family was lower from 6 to 12 h than from 24 to 48 h, whereas the levels of C3H family genes were higher from 6 to 12 h than from 24 to 48 h. The genes of the bZIP and HSF families were abundantly expressed from 6 to 48 h.

### Validation of the DEGs

**Figure 7:**
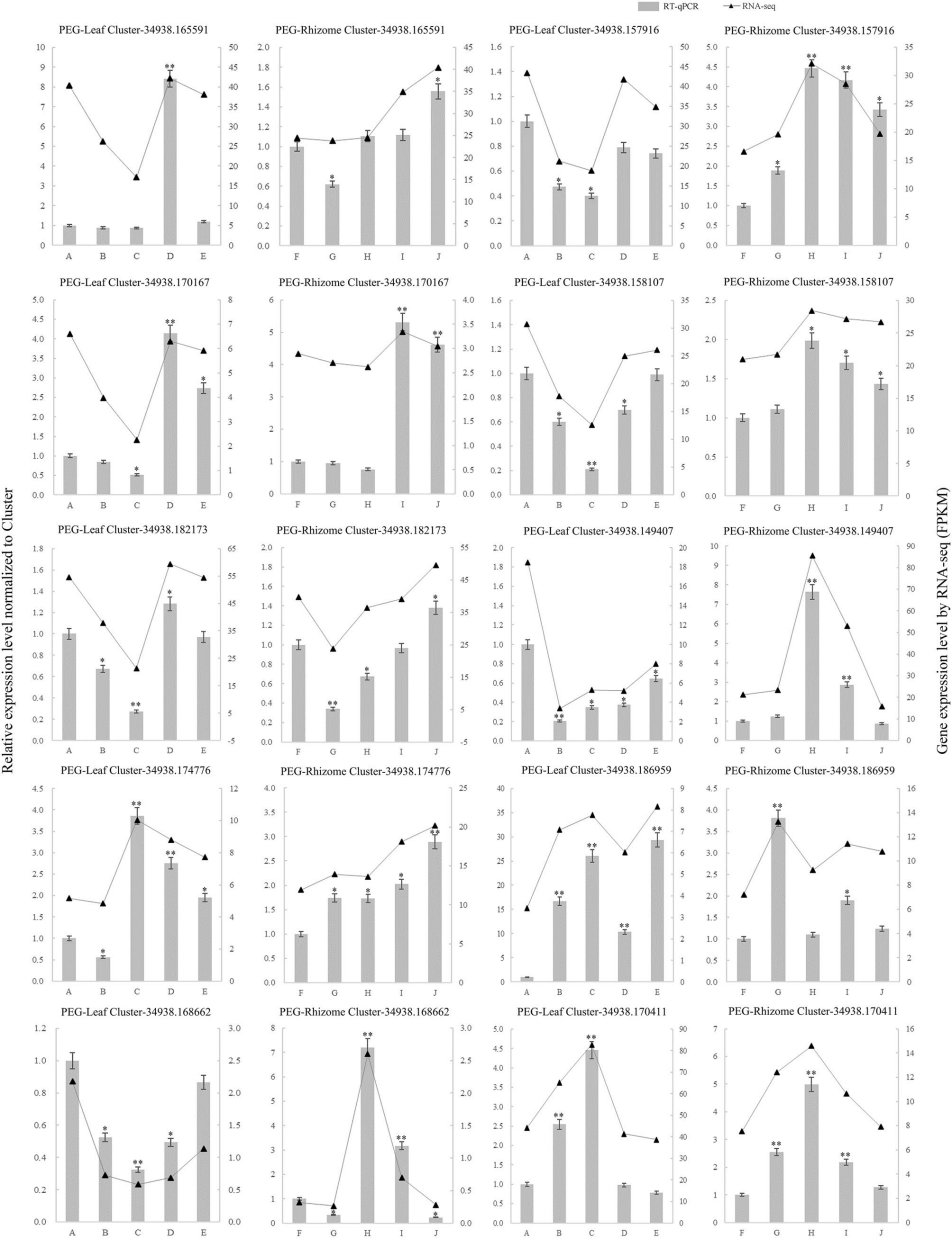
Expression of 10 DEGs in response to drought stress treatment. Vertical bar charts with simple error bars (left y-axis) represent quantitation of 10 genes transcripts in leaf and rhizome samples using RT-qPCR. Values are means ± SE (n = 3). Line graph with triangle (right y-axis) represents transcript abundance (FPKM) of leaf and rhizome samples for each gene detected by RNA-seq. Asterisks (* or **) represent the significant differences at *p* < 0.05 or *p* < 0.01 when compared to the control, respectively (Student’s t-test). The A–E indicates the ‘Little Dream’ of leaves under stress at 0, 6, 12, 24, and 48 h, respectively, the F–J were ‘Little Dream’ of rhizomes under stress at the same time points.

To confirm the reliability of the transcriptome data, we validated the expression levels of 10 candidate DEGs, including five upregulated and five downregulated DEGs involving ARF, WRKY, bZIP, AP2, and other TF families, by RT-qPCR (Supplementary Table S5). The RT-qPCR results were generally in agreement with the results from the transcriptome analysis of the expression patterns of the selected unigenes (Fig. 7).

## Discussion

Drought is one of the most serious natural disasters faced by organisms and significantly influences the growth and development of plants. Under drought stress conditions, plant cell membranes are damaged due to a water deficit, the plant Chl content is decreased, the electrical conductivity is increased, SOD and MDA are rapidly produced, and small molecules, such as SP and Pro, rapidly accumulated^6,21^. In general, plants showing strong drought resistance exhibit smaller changes in their Chl content, a lower electrical conductivity, slower increases in the MDA content, and a small, or relatively stable, decrease in SOD activity^22^. Therefore, these physiological indices can be used for the evaluation of plant drought resistance, which is a complex quantitative characteristic that is affected by environmental and genetic factors. No single index can accurately reflect plant drought resistance, and multiple indices should thus be considered together. The quantitative conversion of experimental data from multiple indicators using the membership function can overcome the limitations of evaluating the drought response using single indices^16^. In this study, we measured the changes in various physiological indexes of 10 *I. germanica* cultivars under drought stress and evaluated the drought resistance of the different cultivars through membership function analysis, which revealed that ‘Little Dream’ exhibited stronger drought resistance than the other cultivars. Bo et al*.* demonstrated that ‘Purple Flower’ and ‘Blood Stone’ are *I. germanica* cultivars with strong drought resistance^16^. In our study, ‘Blood Stone’ also showed strong drought resistance, but ‘Little Dream’ exhibit slightly better performance. This result might be due to regional differences in the selected plant materials and the selected evaluation indices and methods used in the study.

To respond to stress, plants often need both physiological and gene regulation. Understanding the molecular mechanisms underlying the responses to drought stress is important for the establishment of programmes for the breeding of drought-resistant plants. RNA-seq can be used to analyse gene expression and can generate valuable new transcript information for genome-deficient species. The transcriptional stress responses of many plants have been revealed with the development of RNA-seq technology; however, most of these studies have focused on model plants and crops, such as *A. thaliana*^23^, rice^24^, cotton^25^, and soybean^26^, whereas few investigations have explored garden plants. In the present study, the Illumina sequencing platform was used to analyse the response of ‘Little Dream’ to drought stress by the deep sequencing of leaves and rhizomes under simulated drought stress with 20% PEG-6000 solution. From the 30 sequenced sample libraries, 743, 982 unigenes were identified, and this number was higher than those found in previous studies of *Iris* sp.^27^. The read count data were analysed using DESeq2, which identified 31,713 DEGs related to drought stress in ‘Little Dream.’ An approximately threefold higher number of DEGs was identified in rhizomes (24,127) relative to leaves (7849), possibly because rhizomes are directly exposed to external stress conditions and can respond more rapidly to PEG-6000 stress to induce more differential gene expression. Comparisons of the expression patterns in *Atractylodes lancea* leaves and rhizomes also identified more transcripts from rhizomes than from leaves^28^. The number of downregulated genes was greater than that of upregulated genes in rhizomes, which indicated that the expression of many genes was inhibited by drought stress in rhizomes to some extent. The genes identified in both rhizomes and leaves participated in 21 KEGG pathways, which might be important for adaptation to drought stress (Supplementary Table S6). The number of DEGs in leaves and rhizomes changed dynamically in different plant tissues in response to drought stress over time. According to these changes, we categorized the DEGs as long- and short-acting genes and analysed their functions, and the results demonstrated that the two categories had varying functions in different tissues. The early gene functions were mainly concentrated in the processes of gene transcription and translation, which might reflect the preparation of the plants to subsequent physiological changes, whereas the genes expressed later were mainly concentrated in the processes of damage repair and transmembrane ion transport. We divided the DEGs into nine clusters according to their expression patterns and performed a GO analysis to identify the functions enriched in each cluster. The expression patterns and functions showed differences among the clusters, and some clusters showed clear tissue specificity. Our results showed that the response mechanisms of ‘Little Dream’ to drought stress are complex and diverse.

In our study, the ‘ribosome’ pathway exhibited the highest enrichment according to a KEGG analysis and included the largest number of DEGs. Ribosomes are related to protein synthesis because the translation of RNA to protein occurs in ribosomes^29^. One of the main protection mechanisms used by plants in response to drought stress is the ability to renew proteins. Many ribosomal protein genes are differentially regulated in response to various stresses, and the resistance of transgenic plants can be improved by ribosome-related genes^30,31^. We detected 2411 ribosome-related genes, which mainly encoded large and small ribosomal subunit proteins. Only 17 genes were expressed in leaves, and the rest of the genes were expressed in rhizomes (Supplementary Table S7a). This finding might have been obtained because the rhizome of ‘Little Dream’ consists of storage tissues and contains many nutrients, which provide energy for ribosome regulation pathways. Many more ribosome-related DEGs were identified in our transcriptome analysis than in previous studies^32^. Plants can respond to environmental changes through various mechanisms at the transcriptional and translation levels, and ribosomes are plant structures that play important roles in the translation process^33^. This result supports further investigations of drought-guided translation responses and will help researchers obtain a better understanding of the molecular mechanisms involved in the responses of plants to drought stress.

Under drought conditions, plants improve their drought resistance by reducing photosynthesis and transpiration. The KEGG pathway analysis performed in this study showed that photosynthetic antenna proteins and photosynthesis were significantly enriched. In the photosynthetic pathway, most of the genes were upregulated, primarily before the time point of 12 h, and the only exceptions were some downregulated genes related to PSBA, PSBR, and cytochrome b6 (Supplementary Table S7b). The LHC-related genes in the photosynthetic antenna protein pathway also exhibited this characteristic. These findings might reflect the fact that an adequate water supply is stored in the plant at the early stages of drought; hence, the light response is not greatly hindered under conditions with sufficient light. The downregulation of the PSBA gene is consistent with the results from a study of foxtail millet under drought stress^34^. Furthermore, the downregulation of cytochrome B6-related genes demonstrated that photosynthetic pigment synthesis was inhibited to some extent under abiotic stress conditions.

HSPs, which are heat stress-induced proteins that are widely distributed in bacteria, animals, and plants, can help amino acid chains fold into the correct three-dimensional structures and remove and degrade damaged proteins. These proteins protect plant cells from damage by acting as molecular chaperones in response to abiotic stressors, such as high temperature, salinity, drought, and heavy metals^35–37^. HSP20 is the most abundant small HSP in plants. Many HSP20 family genes are induced in rice^38^, soybean^39^, and tomato^40^ under stress conditions, where they potentially play a role in mediating the responses of the plants to environmental stress. In this study, most of the HSP20 genes were upregulated, which indicated that HSP20 might play a positive regulatory role in the response of ‘Little Dream’ to drought stress.

In plants, drought stress can lead to the excessive production of ROS, which are toxic and can damage lipids, proteins, DNA, and carbohydrates^41^. To protect themselves from oxidative stress, plants have evolved antioxidant defence mechanisms. As protective enzymes in plants, SOD, catalase, and peroxidase can effectively scavenge free radicals^42^. In our study, the expression of genes related to SOD, POD and CAT was very abundant, which indicated that these enzymes play an important regulatory role in drought stress. GST can catalyse the binding of nucleophilic glutathione molecules with various electrophilic foreign chemicals, which contributes to detoxification. Overexpression of the *GsGST* gene cloned from wild soybean can enhance the drought and salt tolerance of transgenic tobacco^43^, whereas *LeGSTU2* cloned from tomato can actively improve the tolerance of *A. thaliana* to salt and drought stress^44^. One of the four upregulated GST genes satisfied the criterion |log2 Fold-Change| > 8, which further confirmed that GST is closely related to the drought resistance of *I. germanica*. Ascorbate peroxidase (APX) is an important antioxidant enzyme involved in plant active oxygen metabolism and is the key H_2_O_2_ scavenging enzyme in chloroplasts. Previous studies have shown that APX activity in rice seedlings exposed to PEG was significantly higher than that in the control plants^45^. The expression levels of three l-ascorbate peroxidase genes were all upregulated, which indicated that APX plays a positive role in improving the drought resistance of ‘Little Dream’. These results were consistent with previous findings obtained with *A. thaliana*^46^. Alternative oxidase (AOX) can transfer electrons from UQH2 to O2 via flavoprotein (FP) to produce H2O, which can reduce the formation of mitochondrial ROS in plant cells^47^. The expression of most AOX-related genes was upregulated, which indicated that AOX has an important impact on defences against ROS formation.

Plant hormones are important in regulating plant growth, development, and signal transduction networks. ABA is an important stress hormone that is critical for plant stress responses. Carotenoid cleavage, which is catalysed by NCED, is a key step in regulating ABA biosynthesis^48^. Overexpression of the NCED gene cloned from wilted bean (*Phaseolus vulgaris*) can improve drought resistance in *Nicotiana plumbaginifolia*^49^. We identified three upregulated genes related to NCED, which was consistent with a previous study on foxtail millet^34^, and the results demonstrated that the role of NCED in regulating drought-induced responses was conserved in ‘Little Dream.’ PP2C and SnRK, as part of the ABA signalling pathway, were upregulated in our study, which was consistent with the results from previous studies on *Zea mays* ssp. *Mexicana*^50^. These results suggest that ABA-dependent signal transduction influences the responses of ‘Little Dream’ to drought stress. Auxin is an indispensable hormone in plants. ARFs can specifically bind to the auxin response element of early growth-responsive genes, whereas auxin response protein (AUX/IAA) inhibits ARF activity^51^. SAUR is an early auxin-response gene that is readily induced by exogenous auxin and serves as a negative regulator of auxin synthesis and transport^52^. Ethylene reduces the diffusible auxin levels by inhibiting the synthesis and transport of auxin and thus improves the salt and drought tolerance of plants. Ethylene-responsive TFs of the AP2/ERF family can activate the expression of endogenous genes with a GCC box in their promoter region and thus protect cells from stress^53^. In this study, the upregulated expression of the AP2, IAA and SAUR genes might be due to the inhibition of auxin synthesis by activation of the ethylene biosynthesis pathway during drought stress resistance, and this effect improves the drought resistance of ‘Little Dream.’ The expression of DELLA protein related to GA was upregulated under drought conditions. DELLA protein can inhibit plant growth and improve plant survival under drought stress, which is similar to the results obtained in a study on grapevine (*Vitis vinifera*)^13^. JA and its derivative JAs are important signalling molecules that coordinate plant responses to biotic and abiotic stresses^54^. MYC2 plays an important role in downstream JA-dependent transcriptional reprogramming^55^. MYC2 genes were found to be upregulated in our study, and a similar finding was observed in *Phormium tenax*^56^, which indicates that the MYC2 gene mediates the plant response to drought by regulating JA metabolism. BRs are a unique steroid hormone in plants that can induce the expression of many genes under adverse conditions. Xie et al*.* found that BR signalling regulates the *A. thaliana* response to drought by negatively regulating TINY through BIN2 phosphorylation^57^. In our study, two BIN2 genes were downregulated, but the mechanism through which ‘Little Dream’ resists drought stress needs to be further studied.

As important osmotic substances in plants, the Pro and soluble sugar contents are closely related to drought resistance and accumulate in plants under stress. The overexpression of P5CS, as a key Pro synthase, can improve the tolerance of petunia to water stress^58^. The P5CS-related gene was found to be upregulated in our study, which suggested that ‘Little Dream’ might resist stress-induced damage by regulating Pro synthesis, and this finding was consistent with the results of a study on lotus under cold stress^59^. Trehalose is a rare soluble sugar with a unique ability to protect biomolecules from environmental stress. Increases in or the accumulation of trehalose can improve the resistance of plants to drought and salinity^60^. In our study, TPP, GalAT, PE, UGA4E, and UXS1 were upregulated, which was consistent with the results of a study on *Phormium tenax*^56^. The expression of six TPS genes was downregulated, which differed from the results reported by Bai et al.^56^, possibly due to the diverse trehalose regulation processes in response to drought and the complexity of the regulation mechanism.

Terpenoids, flavonoids, lignins and phenols are important secondary metabolites in plants, and although their physiological significance is not completely clear, these metabolites play specific roles in plant resistance to environmental stress^61^. Terpenoids reportedly determine the terrestrial plant community through allelopathic mechanisms^62^. Flavonoids contain strong antioxidants, which are closely related to the cell ROS content. Under abiotic stress, the flavonoid levels increase in plants, and the overexpression of flavonoid synthesis genes can effectively eliminate ROS and thus improves plant drought resistance^63^. In this study, many genes related to the synthesis of terpenoids and flavonoids were found to be upregulated, which indicated that terpenoids and flavonoids play an active regulatory role in ‘Little Dream’ under drought stress. In addition, we also detected the abundant expression of numerous enzymes related to lignin formation, which indicated that ‘Little Dream’ might promote cell wall modifications by synthesizing lignin to improve its drought resistance under drought conditions. Phenolic compounds are mostly produced through the phenylalanine biosynthesis pathway, and an increase in the phenolic compound content plays an important role in the plant response to stress. Phenylalanine ammonia lyase can catalyse the conversion of l-phenylalanine to trans cinnamic acid and increase its activity under abiotic stress^64,65^. Five upregulated phenylalanine ammonia lyase genes were found in this study, which indicated that phenols might play a key role in the regulation of drought stress.

TFs are important for plant growth and development, morphogenesis, hormone regulation and resistance to a variety of abiotic stressors^66^. In our study, abundant differential regulation of MYB, bHLH, NAC, and WRKY family genes was detected in different tissues during stress. TFs belonging to the MYB family enhance the tolerance of plants to water stress by regulating stomatal movement, scavenging ROS, and participating in stress signal transduction^67^. In *A. thaliana*, *AtMYB12* and *AtMYB30* influence the resistance of the plants to abiotic stress by regulating secondary metabolites and signal transduction^68,69^. In our study, we identified one gene, *IgMYB12* (cluster-34938.230820), that was highly homologous to *AtMYB12* and another gene, *IgMYB30* (cluster-34938.179623), that was homologous to *AtMYB30*. These genes might play key roles in the regulation of drought resistance. Genes belonging to the bHLH family, such as *VvbHLH1* in grape and *PEbHLH35* in *Populus euphratica*^70,71^, have crucial regulatory roles in the responses to drought and salt stress. We identified 25 upregulated bHLH family genes, which indicated that bHLHs play an active role in the responses of ‘Little Dream’ to drought stress. NAC genes comprise a plant-specific TF family. The overexpression of *RhNAC3* in rose petals and *AhNAC3* in peanut can improve plant desiccation and drought tolerance^72,73^, and overexpression of the *NAC13* gene can improve the salt tolerance of poplar trees^74^. In our transcriptome data, 16 NAC-related genes were upregulated, and among these, *IgNAC3* (Cluster-34938.84368) and *IgNAC13* (Cluster-34938.182381, Cluster-34938.87487) might be important in the regulation of drought stress. In addition, *IgNAC14* (Cluster-34938.157299), *IgNAC68* (Cluster-34938.176648), *IgNAC29* (Cluster-34938.187995), and *IgNAC7* (Cluster-34938.84365) might also contribute to drought stress regulation. WRKY TFs have a function in plant defence responses. It has been reported that *AtWRKY33* plays a regulatory role in *A. thaliana* plants under abiotic stress^75^, and we found three upregulated *IgWRKY33* (Cluster-34938.161389, Cluster-34938.159483, and Cluster-34938.171223) genes with high homology to *AtWRKY33* (Supplementary Table S7c). Zn-clus and C2H2 family genes are involved in plant stress responses^76,77^. Overexpression of the C3H family gene *AetTZF1* can improve drought resistance in *A. thaliana*^78^. In this study, Zn-clus, C2H2, and C3H were the three TF families with the largest number of differentially regulated genes, which indicated that these three families are widely involved in the regulation of the responses of ‘Little Dream’ to drought stress. Overall, TFs clearly play crucial roles in the responses of plants to drought stress.

## Conclusion

After the identification of 10 drought-resistant cultivars of *I. germanica* with excellent ornamental characteristics, we screened ‘Little Dream’ as the cultivar exhibiting the strongest resistance to drought stress. Our study provides guidance for the selection of drought-resistant cultivars of garden plants. Furthermore, high-throughput sequencing of leaf and rhizome tissues subjected to simulated drought stress was performed, and this analysis led to the identification of 743,982 unigenes, including 351,551 new transcripts and 31,713 DEGs. Through functional annotation of the DEGs, we identified some important metabolic pathways regulated under drought stress and several key genes potentially involved in the short-term responses to drought. The results of this study constitute the first complete and comprehensive transcriptome map of *I. germanica* under drought stress. This study fills a gap in molecular research on the drought resistance of *I. germanica*. Our findings will strongly promote further research into *Iris* cultivars at the molecular level and enrich the existing genetic information, and thus, the results will provide a valuable genomic resource for the breeding of resistant irises. Our results will also contribute to improvements in the stress tolerance of other iris plants in the future, promote further research on non-model plants, and facilitate the construction of water-saving and drought-resistant gardens.

## Materials and methods

### Plant materials and physiological experiments

The experimental materials were 10 cultivars of *I. germanica* (1 year old rhizome): ‘Cherry Garden,’ ‘Tantara,’ ‘Blood Stone,’ ‘Music Box,’ ‘Memory of Harvest,’ ‘Clarence,’ ‘X’Brassie,’ ‘Little Dream,’ ‘Immortality,’ and ‘White and Gold.’ These cultivars are in full compliance with relevant institutions, national and international norms and legislation. They have excellent ornamental characteristics and are widely used in landscape gardening. To avoid differences caused by variations in provenance, all plant materials were collected from a single nursery in Hebei Province. Peat soil (Pindstrup Sphagnum, Pindstrup Mosebrug A/S, Kongerslev, Denmark) with specifications of 0–6 mm and 10–30 mm was mixed as a planting substrate according to the volume ratio of 1:2(V_0-6 mm_:V_10-30 mm_ = 1:2) and collected in plastic flowerpots (Outside diameter of 17 cm), and the plants were placed in an intelligent greenhouse at Hebei Agricultural University (Baoding, China, 38°49′30″N/115°26ʹ44′E) with an average temperature of 25.3 °C, mean humidity of 53.6%, and light level of approximately 50,000 lx at midday. The plants were taken the same management in all the treatments. In total, 24 individual plants at the vegetative growth stage that exhibited strong and similar growth were selected from each cultivar. All test plants were irrigated for two consecutive days before the experiment to allow the basin soil to fully absorb water and reach saturation. Following irrigation, plants were sampled as controls (under normal water holding conditions) and then again on the 6th, 12th, 18th, 24th, and 30th days after exposure to natural drought. The sampling time was from 8:00 a.m. to 9:00 a.m. The samples were collected from the middle and upper parts of one to two mature leaves next to the centre leaf. Plants from which no leaves were taken were always selected for the experiments. The leaf samples were used for the determination of Chl, MDA, Pro, SP, RP, and SOD activities. Each treatment was repeated three times.

### Transcriptome sequencing experimental design

Based on the results from an evaluation of physiological indices, ‘Little Dream,’ which exhibited the strongest drought resistance, was used as the experimental material for transcriptome sequencing. PEG-6000 is a polymer osmotic agent that is often used to simulate and study plant drought stress^79^. *Iris* species can grow normally under severe (20% PEG-6000) stress. Treatment of the ‘Little Dream’ cultivar with 20% PEG-6000 stress did not cause any obvious damage; therefore, 20% was selected as the concentration of PEG-6000 for simulating drought stress. Various plant tissues respond differently to drought stress; hence, the synchronous sequencing of leaves and rhizomes is beneficial for the mining of drought-resistance genes. The plants were grown in hydroponic containers consisting of transparent plastic basins (400 × 250 × 200 mm) containing Hoagland nutrient solution culture medium. The liquid medium surface was covered with a perforated plastic drift plate, where the pore diameter was dependent on the size of the plant rhizome. Plants of the ‘Little Dream’ strain (*n* = 20) with the same growth status were fixed with a sponge, and one plant was placed in each well. Before the experiment, the plants were incubated in 1/2 Hoagland nutrient solution for 7 days and then transferred to 20% PEG-6000 solution for drought stress treatment. Leaves and rhizomes were collected from the same position at 0, 6, 12, 24, and 48 h. Three replicates of each group were wrapped in tin foil, immediately frozen in liquid nitrogen, and stored at − 80 °C. The treatments were conducted in an artificial climate incubator with a temperature of 25 °C, a relative humidity of approximately 75% and an illumination cycle of 16 h/day.

### Measurement of physiological indices

The leaf Chl content was determined by ethanol extraction^80^. Fresh leaves (0.1 g) were placed in a test tube and extracted in the dark with 10 mL of 95% ethanol for approximately 24 h until the leaves were bleached. Using 95% ethanol as the blank control, the absorbance was determined by spectrophotometry at three wavelengths of 665, 649 and 470 nm to measure the Chl content. The Chl content was calculated using the following formulas: *Ca* (chlorophyll a) = *13.95A*_665_ − *6.88A*_649_; *Cb* (chlorophyll b) = *24.96A*_649_ − *7.32A*_665_; *Cx·c* (carotenoid) = *(1000A*_470_ − *2.05Ca* − *114.8Cb)* × *245*^*–1*^; and chloroplast pigment content (mg g^−1^) = (*C* × *V*_T_ × *n*) × (*FW* × *1000*)^*−1*^, where *A*_665_, *A*_649_ and *A*_470_ are the absorbance values at 665, 649, and 470 nm, respectively, *C* is the concentration of chloroplast pigment (mg L^−1^), *C* = *Ca* + *Cb* + *Cx*·*c*, *FW* is the fresh weight of the sample, *V*_T_ is the total volume of extract, and *n* is the multiple dilution.

The RP was measured using the conductivity method^81^. Fresh leaves (0.2 g) were placed in a test tube, and 20 mL of ultrapure water was added. The tube was shaken vigorously and left to stand at room temperature for 24 h. The electrical conductivity of the solution (*C*_*1*_) was then determined. The solution (including leaves) was bathed at 100 °C for 20 min and cooled to room temperature, and the electrical conductivity (*C*_*2*_) was determined. The following formula was used for the calculation: REC of leaves (%) = *C*_*1*_ × *C*_*2*_^*–1*^ × *100%.*

The SOD activity was determined using the nitrogen blue tetrazole (NBT) reduction method^80^. Fresh leaves (0.2 g) and a small amount of quartz sand were fully ground in 5 mL of phosphate buffer (pH 7.8; 0.05 mol L^−1^) to form a homogenate. The homogenate was centrifuged at 4 °C and 10,000 rpm for 20 min. The supernatant was the enzyme extract. The liquid was then added and determined according to the method described by Zhang et al.^82^, in which the volume of enzyme solution was adjusted to 0.05 mL and the volume of distilled water was adjusted to 0.25 mL. All other conditions were the same as those described by Zhang et al.^82^.

The MDA content was measured using a thiobarbituric acid (TBA) reaction^80^. Fresh leaves (0.1 g) and a small amount of quartz sand were placed in 2 mL of 10% trichloroacetic acid (TCA) and ground to form a homogenate. Subsequently, 8 mL of 10% TCA was added, and the leaves were ground and mixed well. The homogenate was centrifuged at 4 °C and 2000 rpm for 10 min, and the supernatant was the extract. Subsequently, 3 mL of 0.5% TBA was added to 1 mL of extract, and the mixture was mixed well. The reaction was allowed to proceed at 100 °C for 30 min, and the mixture was then cooled immediately and centrifuged for 15 min at 9000 rpm. The supernatant was collected, and TBA solution was used as a blank control. The absorbances at 450, 532, and 600 nm were determined, and the following formula was used for the calculation: MDA content (µmol g^−1^ FW) = *[6.452* × *(D*_532_ − *D*_600_*)* − *0.56D*_450_*]* × *V* × *(W* × *1000)*^*−1*^, where *V* is the volume of the extract.

The SP content was determined by Coomassie Brilliant Blue G-250 staining^83^. The enzyme extract prepared during the determination of SOD activity based on 1 mL was placed in a test tube, and 5 mL of Coomassie Brilliant Blue G-250 solution was then added. The mixture was then fully mixed and incubated for 2 min at room temperature. One millilitre of distilled water was added to 5 mL of Coomassie Brilliant Blue G-250 solution as a control. The absorbance at 595 nm was measured. The standard curve was prepared with bovine serum albumin (BSA). The formula used for the calculation was as follows: soluble protein content (mg g^−1^) = *C* × *VT* × *(VS* × *W* × *1000)*^*−1*^, where *C* is the protein quantity determined from the standard curve (µg), *VT* is the total volume of the extracted solution (mL), *VS* is the volume of the sample solution used for testing (mL), and *W* is the weight of the sample (g).

The accumulation of Pro in leaves was measured using the acid ninhydrin method following the procedure described by Fu et al. with slight modifications^83^. The quantity of fresh leaves used was adjusted to 0.2 g, and the reaction solution was placed away from light for 2 h after the addition of toluene. All other conditions were the same as those described by Fu et al.^83^.

### Total RNA extraction and RNA sequencing

Samples of RNA were extracted from plant leaves and rhizomes following simulated drought treatment for 0, 6, 12, 24, and 48 h. Each treatment was repeated three times, and 30 samples in total were included in the study. Total RNA was isolated using the TRIzol reagent (Life Technologies, Inc., Grand Island, NY, USA) following the manufacturer’s protocol. The degradation and contamination of RNA were evaluated by agarose gel electrophoresis. The purity, concentration, and integrity of RNA were accurately tested using a NanoDrop Qubit 2.0 Fluorometer (Thermo Fisher Scientific, Waltham, MA, USA) and an Agilent 2100 Bioanalyzer (Agilent Technologies, Santa Clara, CA, USA). After the sample quality was confirmed, magnetic beads conjugated with oligo(dT) were used to enrich the mRNA. Subsequently, the mRNA molecules were broken into short 250 bp fragments by adding fragmentation buffer. Using mRNA as the template, first-strand cDNA was synthesized using six-nucleotide random primers. Second-strand cDNA was synthesized by adding buffer, dNTPs, DNA polymerase I, and RNaseH, and the double-stranded cDNA was then purified using AMPure XP beads. The purified double-stranded cDNA was end-filled, subjected to A tail addition and connected to the sequencing label, and the fragments were then size-selected using AMPure XP beads. Finally, quality-controlled cDNA libraries were constructed by polymerase chain reaction (PCR) amplification. Following a quality control procedure, the libraries were paired-end sequenced using the Illumina HiSeq X Ten platform (Novogene, Beijing, China) based on the requirements for effective concentration and targeting of off-machine data. The raw reads were filtered for adapter sequences, and those with an N (undetermined base information) content > 10% and Qphred quality value ≤ 20 (which accounted for more than 50% of all reads) were removed to obtain clean reads. The clean reads were compared with the reference genome of *A. thaliana* for subsequent analysis.

### De novo transcriptome assembly and functional annotation

Because no genome data were available for *I. germanica*, the clean reads were spliced using Trinity to obtain transcript data, and these data were used as a reference for subsequent analyses. The transcript reads and expression patterns were clustered using Corset software (https://code.google.com/p/corset-project/)^84^. To obtain more comprehensive gene function information, the transcripts were annotated by comparison with sequences in the Nr (e-value = 1e^−5^, https://www.ncbi.nlm.nih.gov/), Nt (e-value = 1e^−5^, https://www.ncbi.nlm.nih.gov/), Pfam (e-value = 0.01, http://pfam.sanger.ac.uk/), KOG/COG (e-value = 1e^−3^, http://www.ncbi.nlm.nih.gov/COG/), Swiss-Prot (e-value = 1e^−5^, http://www.ebi.ac.uk/uniprot/), KEGG (e-value = 1e^−10^, http://www.genome.jp/kegg/), and GO (e-value = 1e^−6^, http://www.geneontology.org/) databases. The plant TFs were predicted using iTAK software (http://itak.feilab.net/cgi-bin/itak/index.cgi)^85–87^.

### Identification of gene expression levels and biological analysis of DEGs

The FPKM (number of fragments per million fragments from a gene per kilobase length) method was used for the calculation of gene expression. This method, which is the most commonly used method for estimating gene expression levels, considers the influence of the sequencing depth and gene length on the fragment count^88^. The input data for the analysis of differential gene expression were the read count data obtained from the gene expression level analysis and were analysed using DESeq2^17^. The screening thresholds were padj < 0.05 and |log2 Fold-Change| > 1. The biological functions related to the DEGs and the most important biochemical metabolic pathways and signal transfer pathways related to the DEGs were obtained by GO functional significance^89^ and KEGG (false discovery rate ≤ 0.05) enrichment analyses^90^.

### Quantitative real-time PCR (RT-qPCR)

To verify the reliability of the Illumina RNA-seq results, 10 DEG-encoding proteins from different TF families were validated by RT-qPCR. A 1-μg RNA sample was reverse transcribed using a HiFiScript cDNA Synthesis Kit (CWBIO, Beijing, China). Gene-specific RT-qPCR primers were designed using Primer Premier 5.0 (Premier, Canada) software (Supplementary Table S5). *Tubulin* served as an internal reference gene, and RT-qPCR was performed using Fast Super EvaGreen qPCR Master Mix (US Everbright Inc., Jiangsu, China) and an ABI 7500 Real-Time PCR System (Applied Biosystems, Waltham, MA, USA). The PCR volume was 20 μL, which included 10 μL of 2× Fast Super Eva Green Master Mix, 0.5 μL of the forward primer, 0.5 μL of the reverse primer, 0.2 μL of 10× ROX, 2 μL of the cDNA template, and 6.8 μL of denucleated acid water. The reaction conditions were 2 min at 95 °C for denaturation followed by 45 cycles of 5 s at 95 °C, 5 s at 60 °C, and 50 s at 72 °C. The RT-qPCR analysis consisted of three biological and three technical replications.

### Statistical analysis

The drought resistance of 10 *I. germanica* cultivars was evaluated using the membership function method^82^. The IBM SPSS 21.0 (Armonk, NY, USA) and Microsoft Office Excel 2010 software packages were used to analyse the data from the physiological experiments, and Duncan’s multiple tests was used to evaluate the significance of the differences. *P* < 0.05 considered to indicate statistical significance. The relative expression levels of selected DEGs were calculated using the 2^−ΔΔCt^ method, and independent Student's t test was used to analyze the significance of differences in RT-qPCR results.

## Supplementary Information

Supplementary Figure S1.

Supplementary Figure S2.

Supplementary Figure S3.

Supplementary Figure S4.

Supplementary Table S1.

Supplementary Table S2.

Supplementary Table S3.

Supplementary Table S4.

Supplementary Table S5.

Supplementary Table S6.

Supplementary Table S7.

## Data Availability

The datasets generated during and/or analysed during the current study are available from the corresponding author on reasonable request.
