# Supplementary figures and images for: Evaluation of drought resistance and transcriptome analysis for the identification of drought-responsive genes in Iris germanica
Source: Sci Rep. 2021 Aug 11;11:16308. doi: 10.1038/s41598-021-95633-z (PMC8358056; doi:10.1038/s41598-021-95633-z)

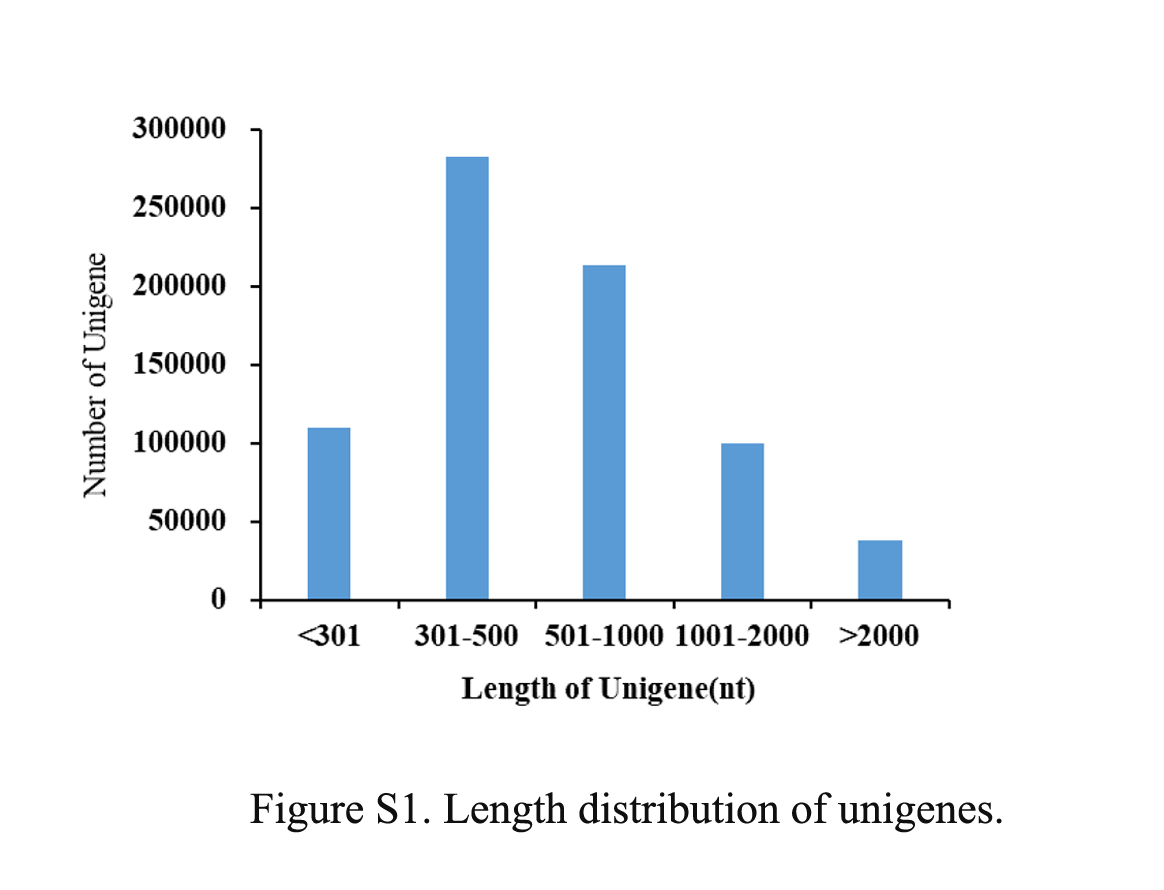

Supplement: Supplementary file 1 — Supplementary Figure S1. [file 41598_2021_95633_MOESM1_ESM.tif]

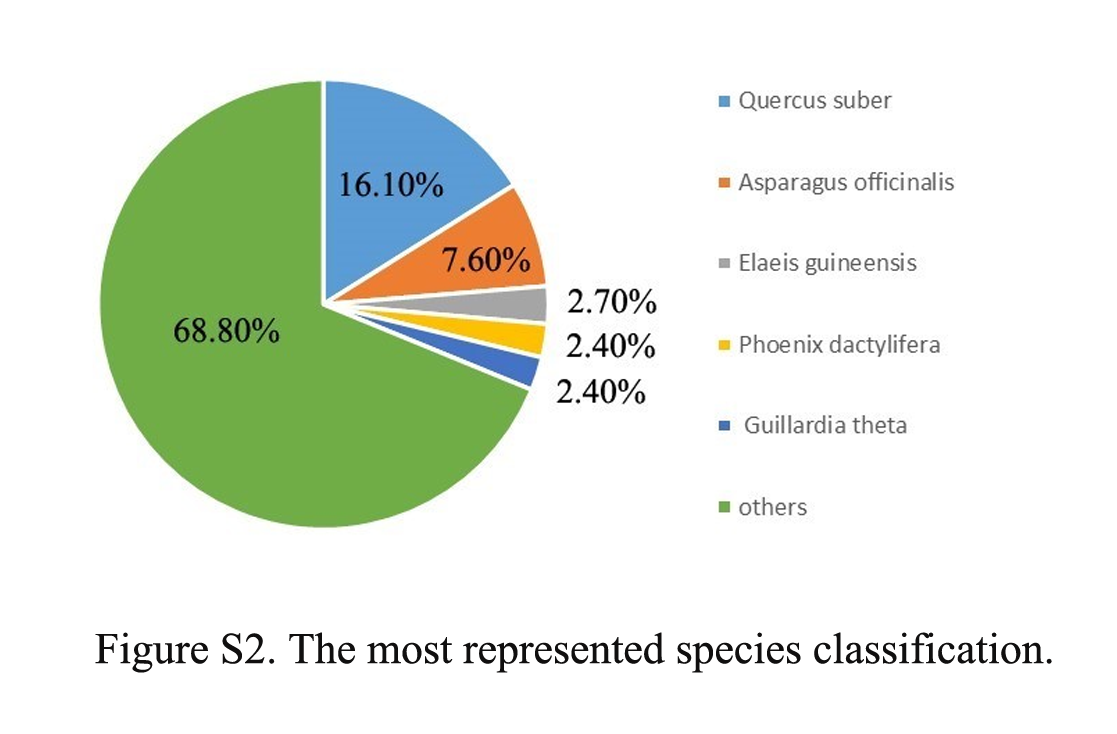

Supplement: Supplementary file 2 — Supplementary Figure S2. [file 41598_2021_95633_MOESM2_ESM.tif]

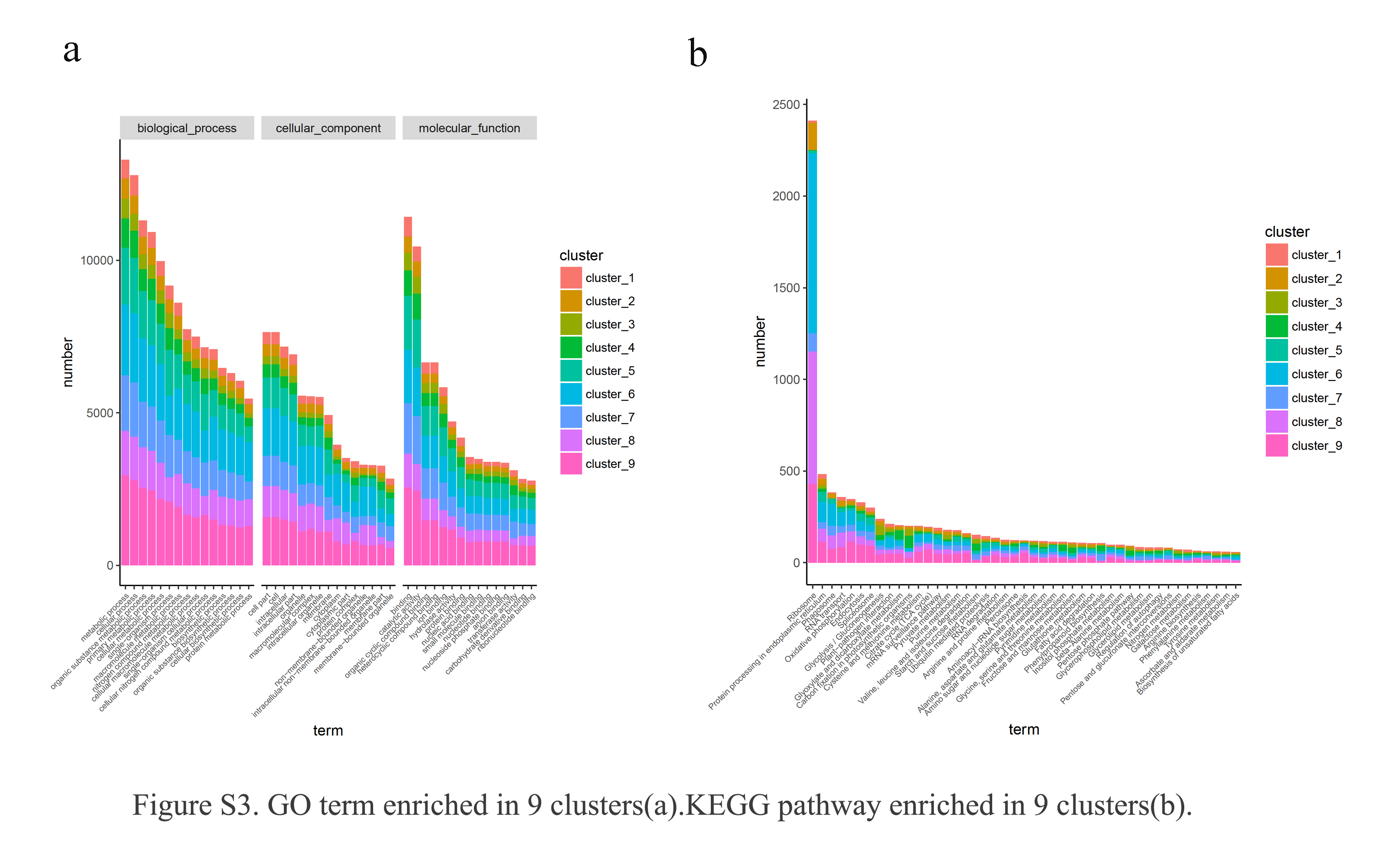

Supplement: Supplementary file 3 — Supplementary Figure S3. [file 41598_2021_95633_MOESM3_ESM.tif]
