# Supplementary material for: Evaluation of drought resistance and transcriptome analysis for the identification of drought-responsive genes in Iris germanica
Source: Sci Rep. 2021 Aug 11;11:16308. doi: 10.1038/s41598-021-95633-z (PMC8358056; doi:10.1038/s41598-021-95633-z)
Supplement: Supplementary file 4 — Supplementary Figure S4. [file 41598_2021_95633_MOESM4_ESM.pdf]

Zn-clus

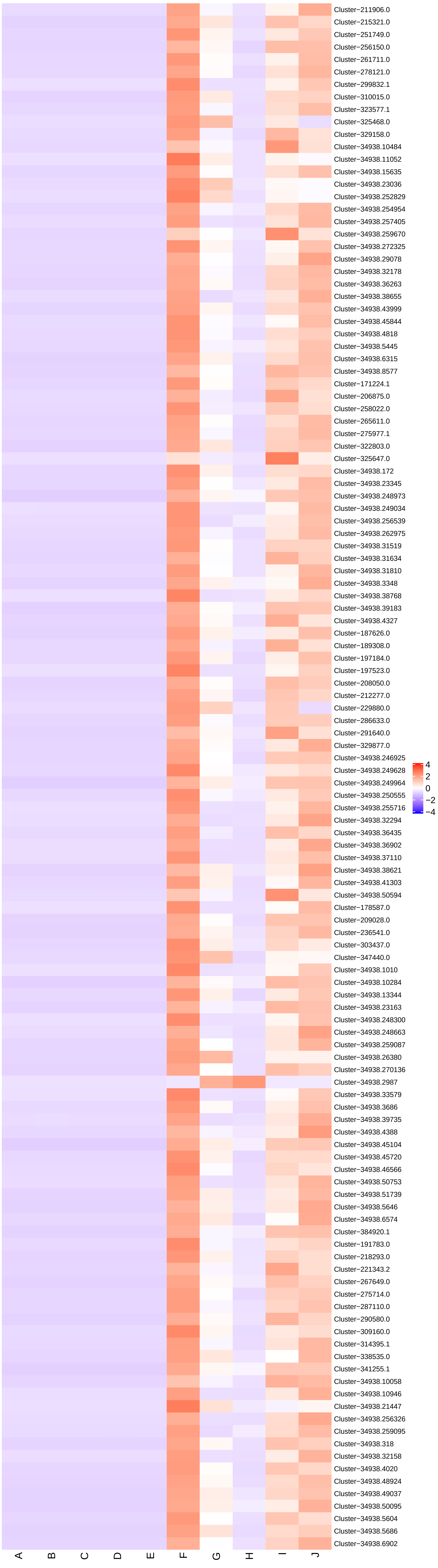
Cluster−211906.0
Cluster−215321.0
Cluster−251749.0
Cluster−256150.0
Cluster−261711.0
Cluster−278121.0
Cluster−299832.1
Cluster−310015.0
Cluster−323577.1
Cluster−325468.0
Cluster−329158.0
Cluster−34938.10484
Cluster−34938.11052
Cluster−34938.15635
Cluster−34938.23036
Cluster−34938.252829
Cluster−34938.254954
Cluster−34938.257405
Cluster−34938.259670
Cluster−34938.272325
Cluster−34938.29078
Cluster−34938.32178
Cluster−34938.36263
Cluster−34938.38655
Cluster−34938.43999
Cluster−34938.45844
Cluster−34938.4818
Cluster−34938.5445
Cluster−34938.6315
Cluster−34938.8577
Cluster−171224.1
Cluster−206875.0
Cluster−258022.0
Cluster−265611.0
Cluster−275977.1
Cluster−322803.0
Cluster−325647.0
Cluster−34938.172
Cluster−34938.23345
Cluster−34938.248973
Cluster−34938.249034
Cluster−34938.256539
Cluster−34938.262975
Cluster−34938.31519
Cluster−34938.31634
Cluster−34938.31810
Cluster−34938.3348
Cluster−34938.38768
Cluster−34938.39183
Cluster−34938.4327
Cluster−187626.0
Cluster−189308.0
Cluster−197184.0
Cluster−197523.0
Cluster−208050.0
Cluster−212277.0
Cluster−229880.0
Cluster−286633.0
Cluster−291640.0
Cluster−329877.0
Cluster−34938.246925
Cluster−34938.249628
Cluster−34938.249964
Cluster−34938.250555
Cluster−34938.255716
Cluster−34938.32294
Cluster−34938.36435
Cluster−34938.36902
Cluster−34938.37110
Cluster−34938.38621
Cluster−34938.41303
Cluster−34938.50594
Cluster−178587.0
Cluster−209028.0
Cluster−236541.0
Cluster−303437.0
Cluster−347440.0
Cluster−34938.1010
Cluster−34938.10284
Cluster−34938.13344
Cluster−34938.23163
Cluster−34938.248300
Cluster−34938.248663
Cluster−34938.259087
Cluster−34938.26380
Cluster−34938.270136
Cluster−34938.2987
Cluster−34938.33579
Cluster−34938.3686
Cluster−34938.39735
Cluster−34938.4388
Cluster−34938.45104
Cluster−34938.45720
Cluster−34938.46566
Cluster−34938.50753
Cluster−34938.51739
Cluster−34938.5646
Cluster−34938.6574
Cluster−384920.1
Cluster−191783.0
Cluster−218293.0
Cluster−221343.2
Cluster−267649.0
Cluster−275714.0
Cluster−287110.0
Cluster−290580.0
Cluster−309160.0
Cluster−314395.1
Cluster−338535.0
Cluster−341255.1
Cluster−34938.10058
Cluster−34938.10946
Cluster−34938.21447
Cluster−34938.256326
Cluster−34938.259095
Cluster−34938.318
Cluster−34938.32158
Cluster−34938.4020
Cluster−34938.48924
Cluster−34938.49037
Cluster−34938.50095
Cluster−34938.5604
Cluster−34938.5686
Cluster−34938.6902
4
2
0
−2
−4
A
B
C
D
E
F
G
H
I
J

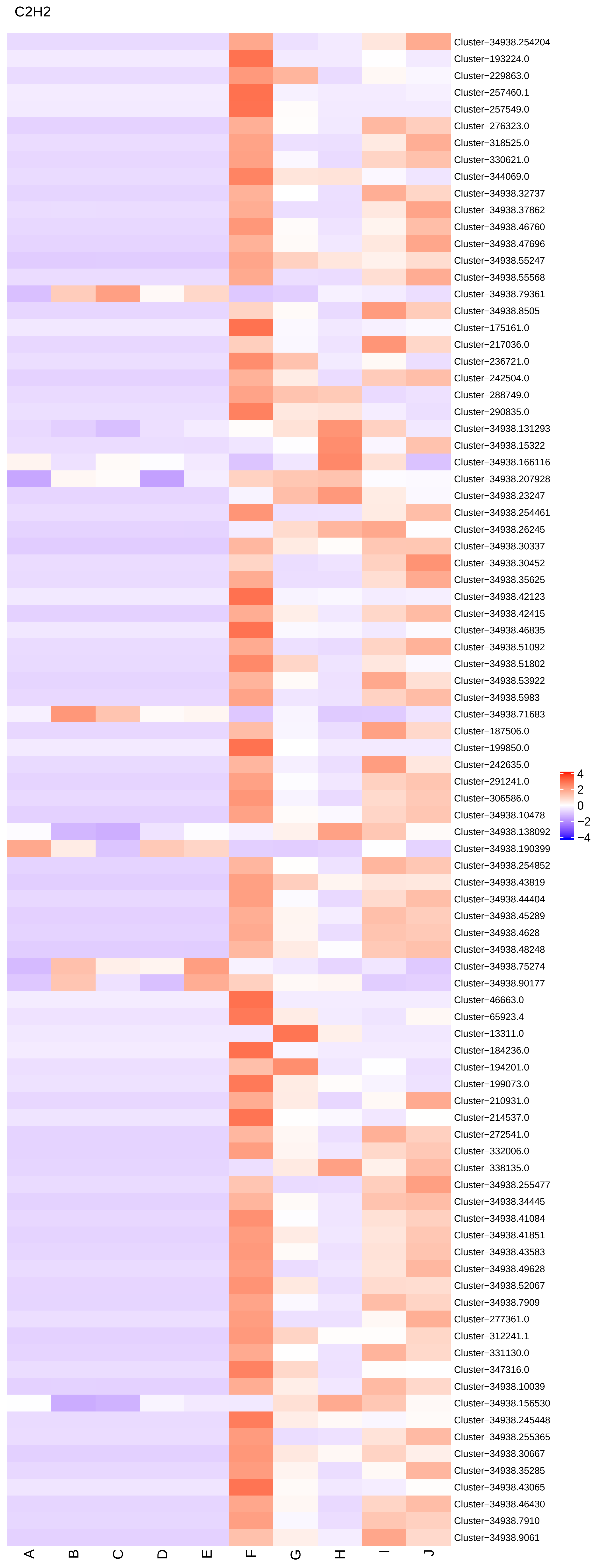
C2H2
Cluster−34938.254204
Cluster−193224.0
Cluster−229863.0
Cluster−257460.1
Cluster−257549.0
Cluster−276323.0
Cluster−318525.0
Cluster−330621.0
Cluster−344069.0
Cluster−34938.32737
Cluster−34938.37862
Cluster−34938.46760
Cluster−34938.47696
Cluster−34938.55247
Cluster−34938.55568
Cluster−34938.79361
Cluster−34938.8505
Cluster−175161.0
Cluster−217036.0
Cluster−236721.0
Cluster−242504.0
Cluster−288749.0
Cluster−290835.0
Cluster−34938.131293
Cluster−34938.15322
Cluster−34938.166116
Cluster−34938.207928
Cluster−34938.23247
Cluster−34938.254461
Cluster−34938.26245
Cluster−34938.30337
Cluster−34938.30452
Cluster−34938.35625
Cluster−34938.42123
Cluster−34938.42415
Cluster−34938.46835
Cluster−34938.51092
Cluster−34938.51802
Cluster−34938.53922
Cluster−34938.5983
Cluster−34938.71683
Cluster−187506.0
Cluster−199850.0
Cluster−242635.0
Cluster−291241.0
Cluster−306586.0
Cluster−34938.10478
Cluster−34938.138092
Cluster−34938.190399
Cluster−34938.254852
Cluster−34938.43819
Cluster−34938.44404
Cluster−34938.45289
Cluster−34938.4628
Cluster−34938.48248
Cluster−34938.75274
Cluster−34938.90177
Cluster−46663.0
Cluster−65923.4
Cluster−13311.0
Cluster−184236.0
Cluster−194201.0
Cluster−199073.0
Cluster−210931.0
Cluster−214537.0
Cluster−272541.0
Cluster−332006.0
Cluster−338135.0
Cluster−34938.255477
Cluster−34938.34445
Cluster−34938.41084
Cluster−34938.41851
Cluster−34938.43583
Cluster−34938.49628
Cluster−34938.52067
Cluster−34938.7909
Cluster−277361.0
Cluster−312241.1
Cluster−331130.0
Cluster−347316.0
Cluster−34938.10039
Cluster−34938.156530
Cluster−34938.245448
Cluster−34938.255365
Cluster−34938.30667
Cluster−34938.35285
Cluster−34938.43065
Cluster−34938.46430
Cluster−34938.7910
Cluster−34938.9061
A
B
C
D
E
F
G
H
I
J
4
2
0
−2
−4

C3H

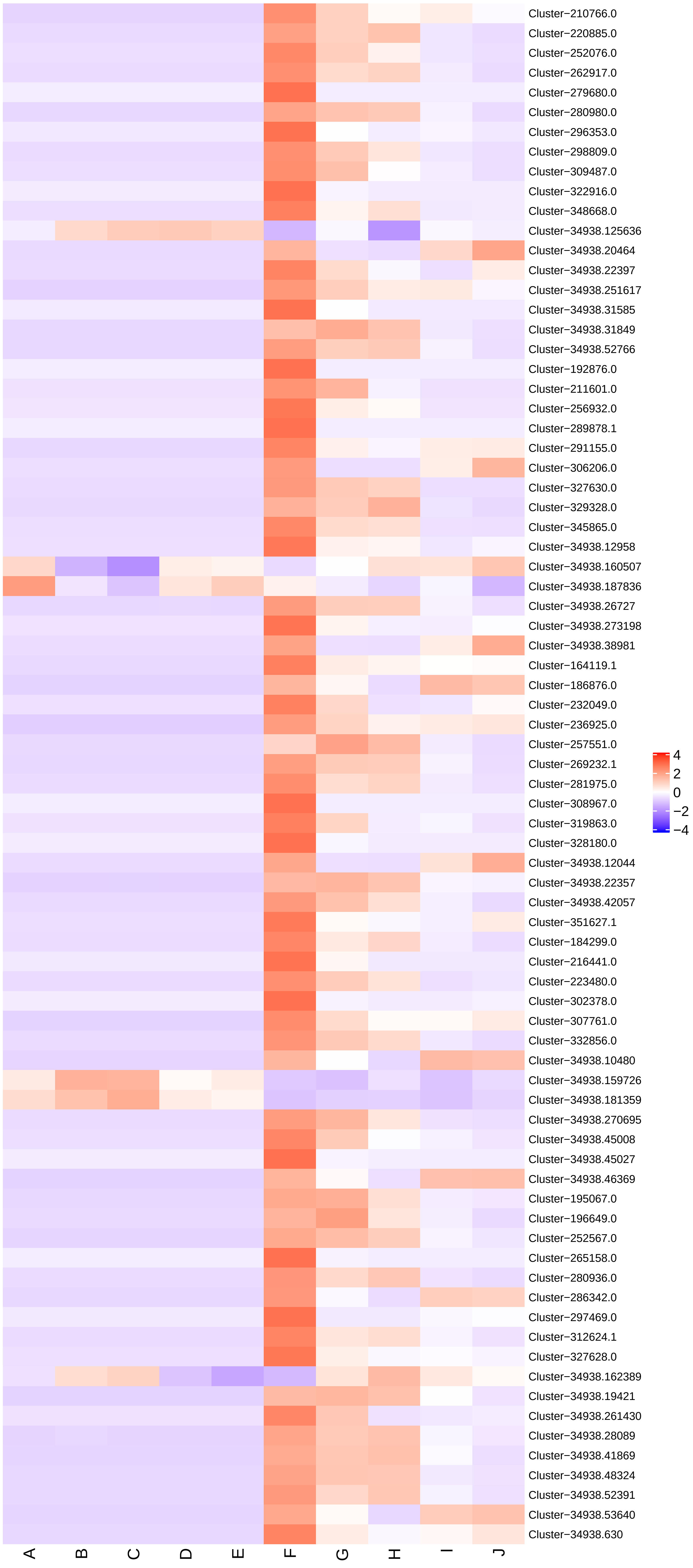
Cluster−210766.0
Cluster−220885.0
Cluster−252076.0
Cluster−262917.0
Cluster−279680.0
Cluster−280980.0
Cluster−296353.0
Cluster−298809.0
Cluster−309487.0
Cluster−322916.0
Cluster−348668.0
Cluster−34938.125636
Cluster−34938.20464
Cluster−34938.22397
Cluster−34938.251617
Cluster−34938.31585
Cluster−34938.31849
Cluster−34938.52766
Cluster−192876.0
Cluster−211601.0
Cluster−256932.0
Cluster−289878.1
Cluster−291155.0
Cluster−306206.0
Cluster−327630.0
Cluster−329328.0
Cluster−345865.0
Cluster−34938.12958
Cluster−34938.160507
Cluster−34938.187836
Cluster−34938.26727
Cluster−34938.273198
Cluster−34938.38981
Cluster−164119.1
Cluster−186876.0
Cluster−232049.0
Cluster−236925.0
Cluster−257551.0
Cluster−269232.1
Cluster−281975.0
Cluster−308967.0
Cluster−319863.0
Cluster−328180.0
Cluster−34938.12044
Cluster−34938.22357
Cluster−34938.42057
Cluster−351627.1
Cluster−184299.0
Cluster−216441.0
Cluster−223480.0
Cluster−302378.0
Cluster−307761.0
Cluster−332856.0
Cluster−34938.10480
Cluster−34938.159726
Cluster−34938.181359
Cluster−34938.270695
Cluster−34938.45008
Cluster−34938.45027
Cluster−34938.46369
Cluster−195067.0
Cluster−196649.0
Cluster−252567.0
Cluster−265158.0
Cluster−280936.0
Cluster−286342.0
Cluster−297469.0
Cluster−312624.1
Cluster−327628.0
Cluster−34938.162389
Cluster−34938.19421
Cluster−34938.261430
Cluster−34938.28089
Cluster−34938.41869
Cluster−34938.48324
Cluster−34938.52391
Cluster−34938.53640
Cluster−34938.630
4
2
0
−2
−4
A
B
C
D
E
F
G
H
I
J

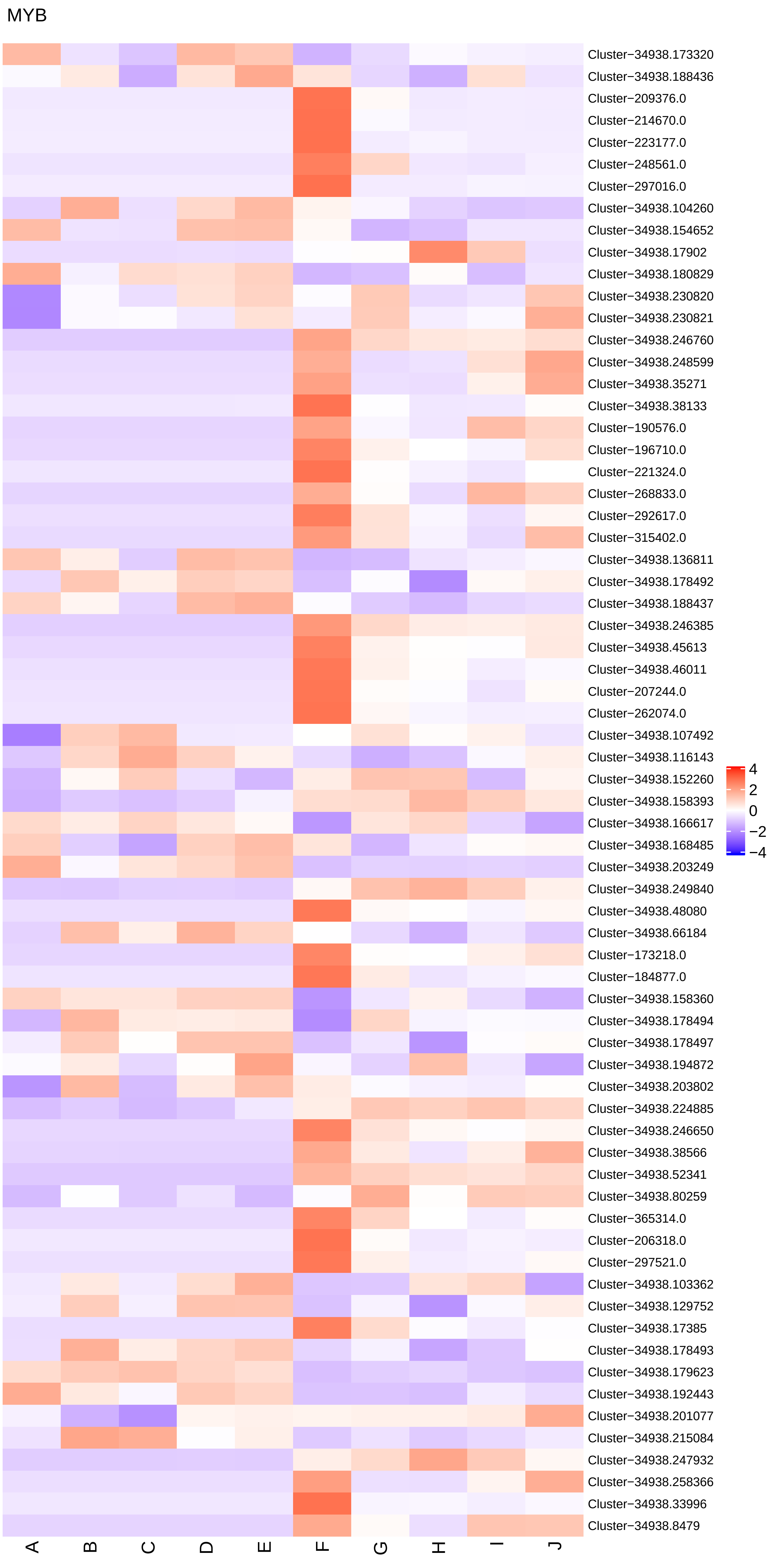
MYB
Cluster−34938.173320
Cluster−34938.188436
Cluster−209376.0
Cluster−214670.0
Cluster−223177.0
Cluster−248561.0
Cluster−297016.0
Cluster−34938.104260
Cluster−34938.154652
Cluster−34938.17902
Cluster−34938.180829
Cluster−34938.230820
Cluster−34938.230821
Cluster−34938.246760
Cluster−34938.248599
Cluster−34938.35271
Cluster−34938.38133
Cluster−190576.0
Cluster−196710.0
Cluster−221324.0
Cluster−268833.0
Cluster−292617.0
Cluster−315402.0
Cluster−34938.136811
Cluster−34938.178492
Cluster−34938.188437
Cluster−34938.246385
Cluster−34938.45613
Cluster−34938.46011
Cluster−207244.0
Cluster−262074.0
Cluster−34938.107492
Cluster−34938.116143
Cluster−34938.152260
Cluster−34938.158393
Cluster−34938.166617
Cluster−34938.168485
Cluster−34938.203249
Cluster−34938.249840
Cluster−34938.48080
Cluster−34938.66184
Cluster−173218.0
Cluster−184877.0
Cluster−34938.158360
Cluster−34938.178494
Cluster−34938.178497
Cluster−34938.194872
Cluster−34938.203802
Cluster−34938.224885
Cluster−34938.246650
Cluster−34938.38566
Cluster−34938.52341
Cluster−34938.80259
Cluster−365314.0
Cluster−206318.0
Cluster−297521.0
Cluster−34938.103362
Cluster−34938.129752
Cluster−34938.17385
Cluster−34938.178493
Cluster−34938.179623
Cluster−34938.192443
Cluster−34938.201077
Cluster−34938.215084
Cluster−34938.247932
Cluster−34938.258366
Cluster−34938.33996
Cluster−34938.8479
4
2
0
−2
−4
A
B
C
D
E
F
G
H
I
J

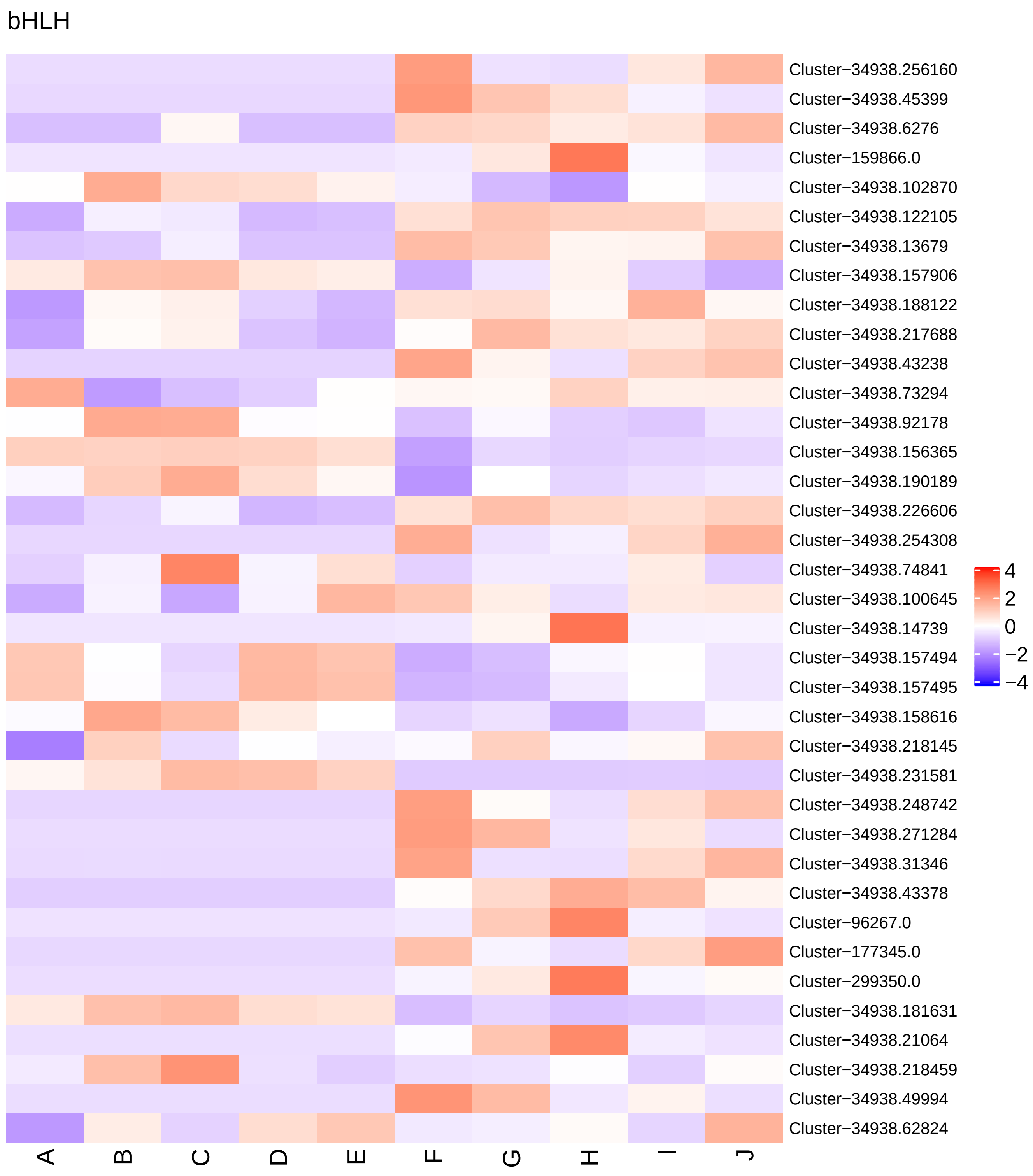
bHLH
Cluster−34938.256160
Cluster−34938.45399
Cluster−34938.6276
Cluster−159866.0
Cluster−34938.102870
Cluster−34938.122105
Cluster−34938.13679
Cluster−34938.157906
Cluster−34938.188122
Cluster−34938.217688
Cluster−34938.43238
Cluster−34938.73294
Cluster−34938.92178
Cluster−34938.156365
Cluster−34938.190189
Cluster−34938.226606
Cluster−34938.254308
Cluster−34938.74841
Cluster−34938.100645
Cluster−34938.14739
Cluster−34938.157494
Cluster−34938.157495
Cluster−34938.158616
Cluster−34938.218145
Cluster−34938.231581
Cluster−34938.248742
Cluster−34938.271284
Cluster−34938.31346
Cluster−34938.43378
Cluster−96267.0
Cluster−177345.0
Cluster−299350.0
Cluster−34938.181631
Cluster−34938.21064
Cluster−34938.218459
Cluster−34938.49994
Cluster−34938.62824
4
2
0
−2
−4
A
B
C
D
E
F
G
H
I
J

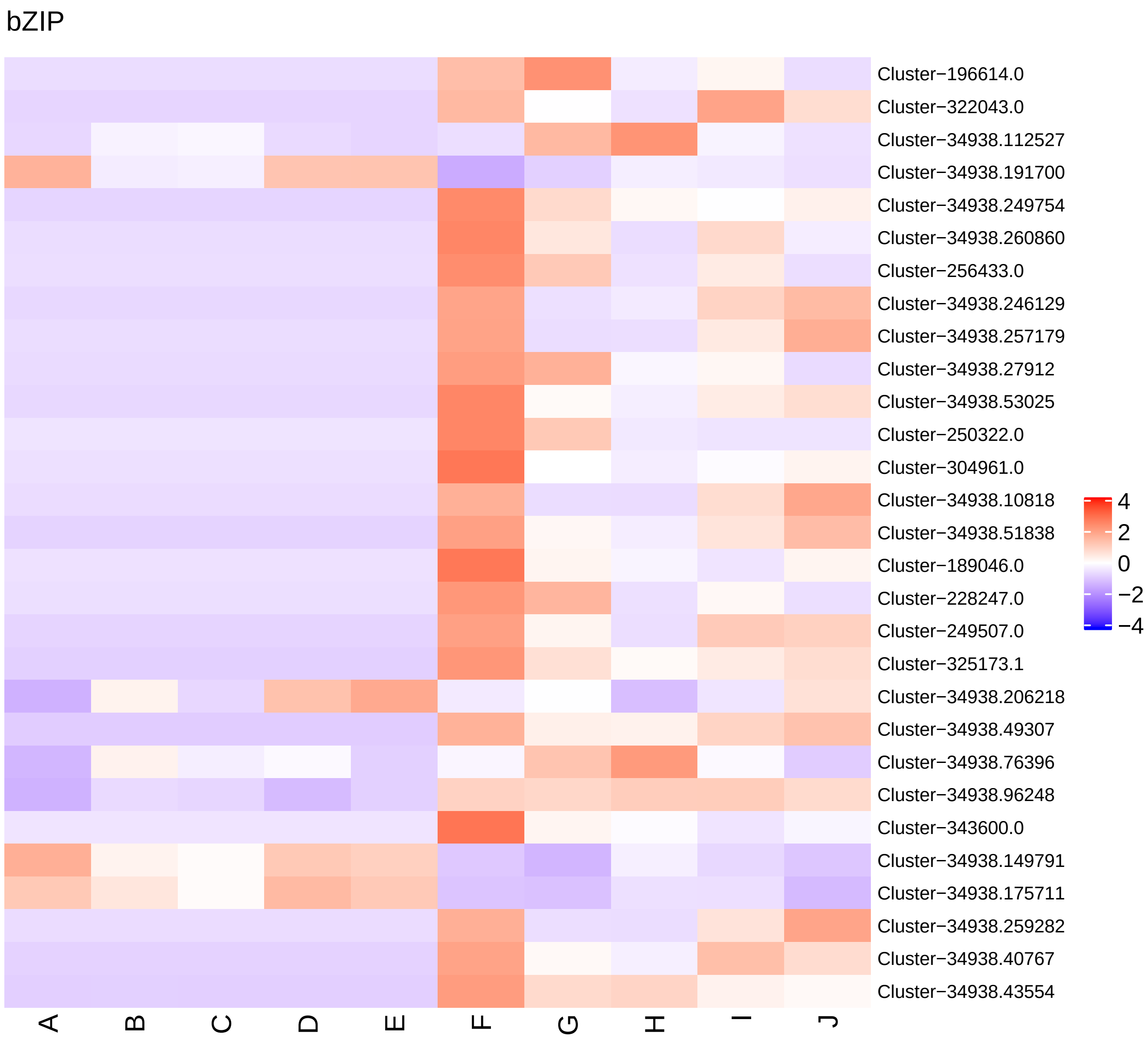
bZIP
Cluster−196614.0
Cluster−322043.0
Cluster−34938.112527
Cluster−34938.191700
Cluster−34938.249754
Cluster−34938.260860
Cluster−256433.0
Cluster−34938.246129
Cluster−34938.257179
Cluster−34938.27912
Cluster−34938.53025
Cluster−250322.0
Cluster−304961.0
Cluster−34938.10818
Cluster−34938.51838
Cluster−189046.0
Cluster−228247.0
Cluster−249507.0
Cluster−325173.1
Cluster−34938.206218
Cluster−34938.49307
Cluster−34938.76396
Cluster−34938.96248
Cluster−343600.0
Cluster−34938.149791
Cluster−34938.175711
Cluster−34938.259282
Cluster−34938.40767
Cluster−34938.43554
A
B
C
D
E
F
G
H
I
J
4
2
0
−2
−4

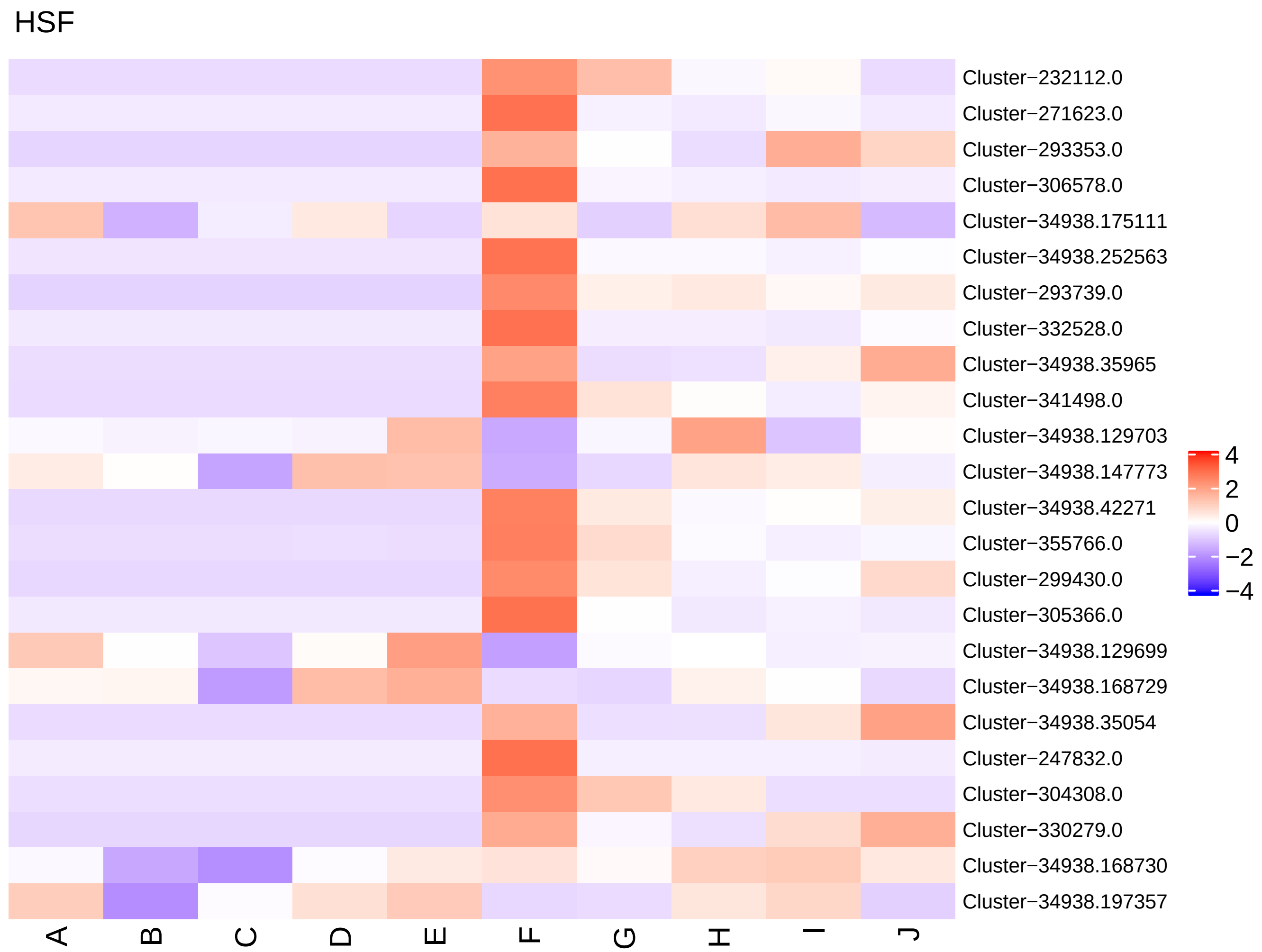
HSF
Cluster−232112.0
Cluster−271623.0
Cluster−293353.0
Cluster−306578.0
Cluster−34938.175111
Cluster−34938.252563
Cluster−293739.0
Cluster−332528.0
Cluster−34938.35965
Cluster−341498.0
Cluster−34938.129703
Cluster−34938.147773
Cluster−34938.42271
Cluster−355766.0
Cluster−299430.0
Cluster−305366.0
Cluster−34938.129699
Cluster−34938.168729
Cluster−34938.35054
Cluster−247832.0
Cluster−304308.0
Cluster−330279.0
Cluster−34938.168730
Cluster−34938.197357
A
B
C
D
E
F
G
H
I
J
4
2
0
−2
−4

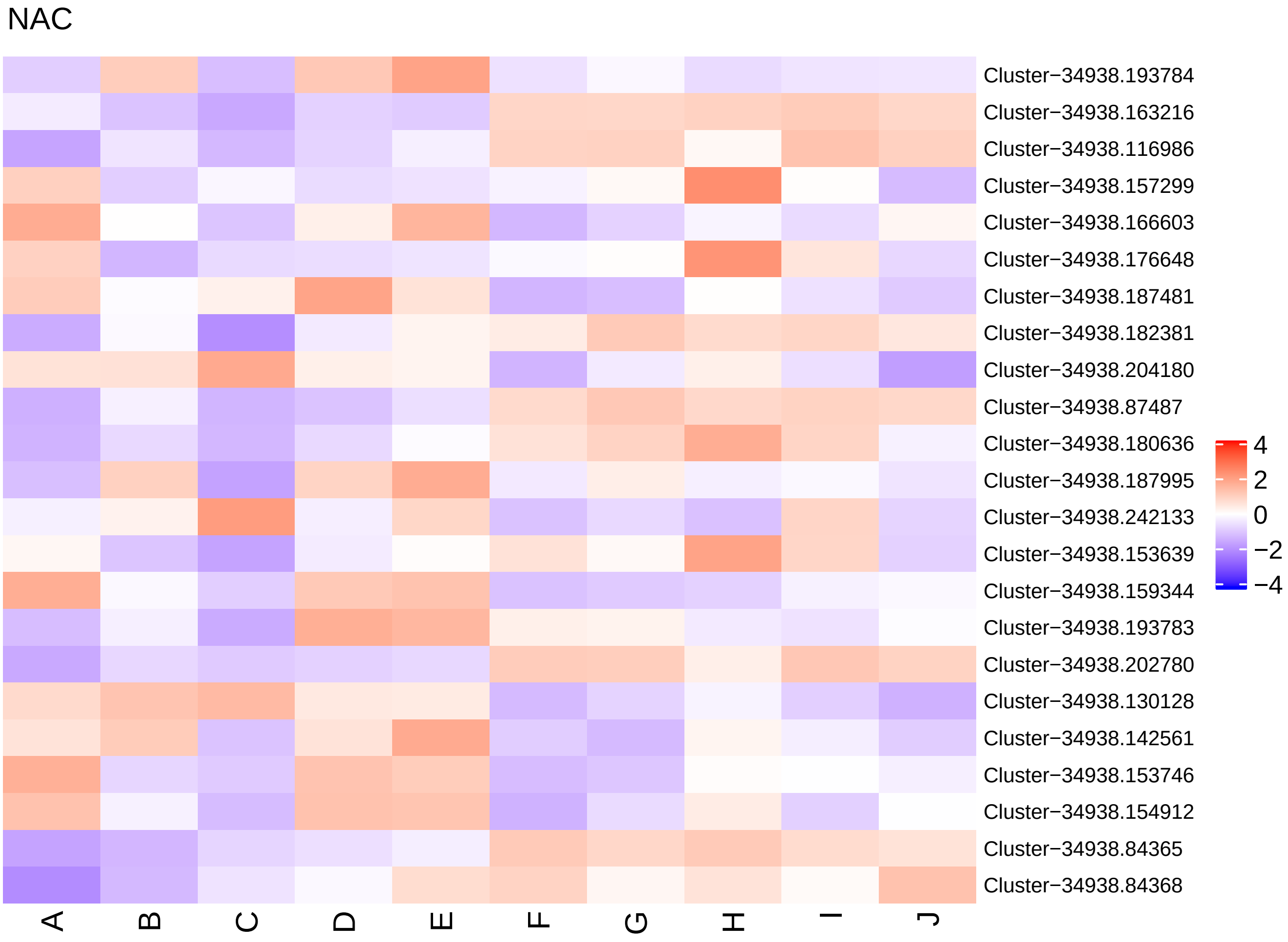
NAC
Cluster−34938.193784
Cluster−34938.163216
Cluster−34938.116986
Cluster−34938.157299
Cluster−34938.166603
Cluster−34938.176648
Cluster−34938.187481
Cluster−34938.182381
Cluster−34938.204180
Cluster−34938.87487
Cluster−34938.180636
Cluster−34938.187995
Cluster−34938.242133
Cluster−34938.153639
Cluster−34938.159344
Cluster−34938.193783
Cluster−34938.202780
Cluster−34938.130128
Cluster−34938.142561
Cluster−34938.153746
Cluster−34938.154912
Cluster−34938.84365
Cluster−34938.84368
A
B
C
D
E
F
G
H
I
J
4
2
0
−2
−4

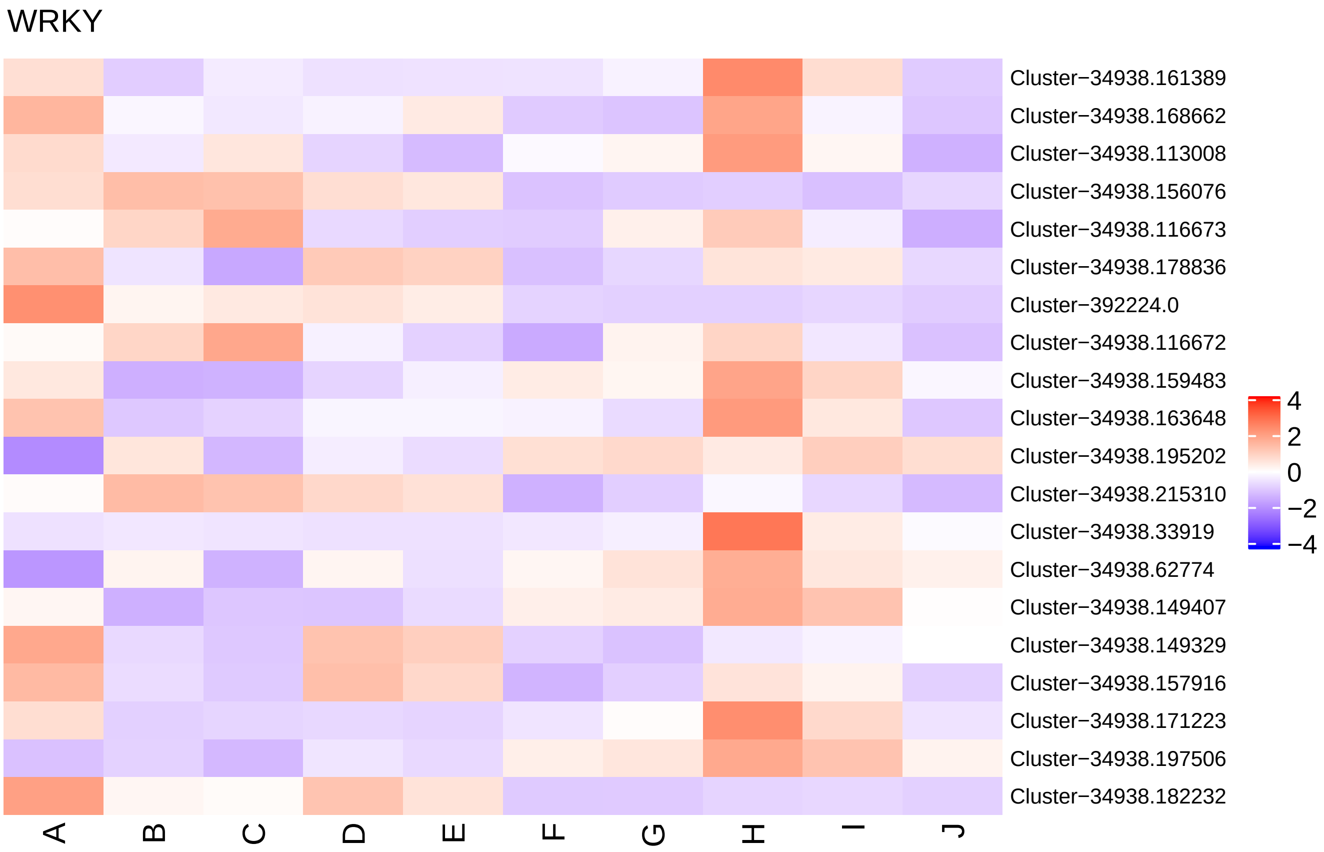

Figure S4. Expression patterns of transcription factors in leaf and rhizome samples.
