# Supplementary material for: Evaluation of drought resistance and transcriptome analysis for the identification of drought-responsive genes in Iris germanica
Source: Sci Rep. 2021 Aug 11;11:16308. doi: 10.1038/s41598-021-95633-z (PMC8358056; doi:10.1038/s41598-021-95633-z)
Supplement: Supplementary file 5 — Supplementary Table S1. [file 41598_2021_95633_MOESM5_ESM.docx]

**Supplementary Table S1.** Changs in physiological indexes in response to drought stress in *I. germanica*

1. The Chl content of *I. germanica* leaves at different stress times (mg·g^-1^ FW)

| Chl content  Cultivars | Days of drought stress/ days | | | | | |
| --- | --- | --- | --- | --- | --- | --- |
|  | 0 | 6 | 12 | 18 | 24 | 30 |
| Cherry Garden | 1.07±0.71abc | 2.47±0.04a | 1.24±0.24a | 1.07±0.12bcd | 0.84±0.02abc | 1.53±0.20a |
| Tantara | 1.53±0.41a | 1.94±0.44a | 1.06±0.31ab | 1.38±0.02ab | 0.66±0.05bcd | 0.24±0.10e |
| Blood Stone | 0.53±0.07bc | 2.58±0.09a | 0.89±0.27ab | 0.58±0.05ef | 0.44±0.18d | 0.95±0.08bcd |
| Music Box | 0.52±0.05bc | 2.31±0.28a | 1.07±0.25ab | 0.87±0.06de | 0.79±0.09abcd | 1.18±0.23ab |
| Memory of Harvest | 0.29±0.07c | 1.97±0.37a | 0.57±0.31b | 1.57±0.23a | 1.12±0.13a | 0.88±0.33bcd |
| Clarence | 0.54±0.23bc | 1.96±0.45a | 1.17±0.05a | 1.31±0.35abc | 1.01±0.21ab | 0.70±0.04cd |
| X’Brassie | 0.83±0.42abc | 1.82±0.35a | 1.05±0.20ab | 0.73±0.14de | 0.59±0.23cd | 0.65±0.11d |
| Little Dream | 0.75±0.06abc | 2.16±0.22a | 0.92±0.05ab | 0.95±0.28cde | 0.75±0.10bcd | 0.72±0.27cd |
| Immortality | 0.77±0.46abc | 2.49±0.03a | 0.96±0.15ab | 1.34±0.08abc | 0.86±0.18abc | 1.11±0.17bc |
| White and Gold | 1.23±0.30ab | 2.08±0.55a | 1.02±0.05ab | 0.25±0.13f | 0.46±0.17d | 0.75±0.15bcd |

The results of physiological indexes were expressed as mean±standard deviation, different letters between different cultivar at the same time point indicate significant differences at P<0.05 levels through Duncan’s multiple test, the same below.

1. The REC of *I. germanica* leaves at different stress times (%)

| REC  Cultivars | Days of drought stress/ days | | | | | |
| --- | --- | --- | --- | --- | --- | --- |
|  | 0 | 6 | 12 | 18 | 24 | 30 |
| Cherry Garden | 13.35±0.94c | 14.36±2.71c | 19.06±0.76cd | 20.62±2.03cd | 30.71±3.14d | 37.45±0.66d |
| Tantara | 10.37±1.05c | 15.53±1.15c | 15.55±0.54d | 18.38±0.75d | 26.72±1.02e | 32.27±0.74e |
| Blood Stone | 22.46±0.53b | 26.33±0.91b | 31.69±0.64b | 40.56±0.68b | 50.39±0.74b | 64.71±0.64a |
| Music Box | 12.23±1.07c | 18.55±0.97c | 19.79±3.38c | 24.19±2.34c | 34.76±0.75c | 42.31±1.47c |
| Memory of Harvest | 20.94±1.11b | 32.62±2.33a | 41.19±2.53a | 48.98±2.08a | 55.14±1.42a | 64.43±3.37a |
| Clarence | 12.16±1.82c | 17.83±4.41c | 18.65±1.28cd | 21.26±3.63cd | 29.22±0.72de | 35.09±2.63de |
| X’Brassie | 21.80±1.14b | 26.06±1.05b | 32.11±1.04b | 40.55±0.68b | 50.54±0.88b | 66.62±0.97a |
| Little Dream | 27.31±1.35a | 29.87±0.67ab | 37.37±0.68a | 45.11±1.19ab | 49.27±2.36b | 51.89±1.48b |
| Immortality | 10.69±0.93c | 14.71±0.87c | 18.91±0.34cd | 22.89±3.35cd | 32.42±2.21cd | 38.32±3.24d |
| White and Gold | 21.83±2.37b | 31.59±2.85a | 40.73±3.08a | 47.93±1.65a | 57.04±2.36a | 65.45±0.61a |

1. The SOD activities of *I. germanica* leaves at different stress times (U·g^-1^FW)

| SOD activity  Cultivars | Days of drought stress/ days | | | | | |
| --- | --- | --- | --- | --- | --- | --- |
|  | 0 | 6 | 12 | 18 | 24 | 30 |
| Cherry Garden | 805.85±22.18a | 892.77±276.91a | 579.09±94.13ab | 360.61±76.92d | 411.02±25.42c | 527.78±159.91abc |
| Tantara | 493.74±34.94cd | 171.91±36.63d | 352.55±27.37bc | 100.08±15.38e | 434.32±150.42c | 550.68±73.41bcd |
| Blood Stone | 764.09±104.21a | 637.53±249.41ab | 368.63±94.33bc | 624.05±88.24cd | 406.31±75.61c | 844.21±161.67a |
| Music Box | 791.23±151.79ab | 473.19±276.91abcd | 441.73±156.63abc | 486.41±85.24bcd | 449.03±38.09c | 696.69±66.12ab |
| Memory of Harvest | 301.67±86.45d | 187.57±63.36d | 340.88±67.53abc | 825.51±152.61ab | 515.07±25.72bc | 576.80±117.28bc |
| Clarence | 449.90±58.08cd | 210.37±31.88cd | 462.47±146.06abc | 389.13±91.40cd | 541.82±32.93bc | 443.86±40.32cd |
| X’Brassie | 755.74±39.25ab | 539.63±121.53ab | 617.96±175.07abc | 717.01±151.71bc | 432.77±78.69c | 672.51±84.80ab |
| Little Dream | 811.06±58.56a | 807.69±310.41abc | 415.55±94.13abc | 601.17±81.69b | 530.37±170.64ab | 702.58±94.68ab |
| Immortality | 611.69±78.11bc | 289.74±93.52bcd | 868.63±288.23a | 906.16±115.17a | 680.79±148.31ab | 357.33±67.57d |
| White and Gold | 726.51±50.11ab | 713.29±210.49abcd | 189.01±27.02c | 412.02±158.09bcd | 753.77±89.69a | 614.04±110.53bc |

1. The MDA content of *I. germanica* leaves at different stress times (nmol·g^-1^ FW)

| MDA content  Cultivars | Days of drought stress/ days | | | | | |
| --- | --- | --- | --- | --- | --- | --- |
|  | 0 | 6 | 12 | 18 | 24 | 30 |
| Cherry Garden | 20.87±3.76b | 13.42±3.61bc | 21.72±5.10a | 16.55±6.22a | 24.14±2.64ab | 28.33±0.82b |
| Tantara | 28.29±6.25a | 12.70±0.51bc | 19.53±8.23a | 26.90±7.04a | 19.41±0.63bcd | 24.70±0.82bc |
| Blood Stone | 18.37±1.67bc | 13.25±2.80bc | 33.08±8.94a | 20.15±3.12a | 20.87±1.14abcd | 21.83±1.28cd |
| Music Box | 16.76±3.84bc | 14.15±1.76bc | 20.66±2.75a | 22.34±3.54a | 25.25±2.70a | 28.06±3.63b |
| Memory of Harvest | 16.49±1.98bc | 7.34±0.41c | 24.47±7.17a | 15.88±3.86a | 20.80±1.95abcd | 20.36±0.94de |
| Clarence | 14.41±0.28bc | 11.20±1.39bc | 20.20±3.15a | 21.59±7.93a | 22.63±2.65ab | 28.39±0.71b |
| X’Brassie | 13.73±2.11c | 21.17±6.48a | 24.98±4.95a | 23.80±10.73a | 22.21±3.25abc | 21.30±2.14cd |
| Little Dream | 19.99±2.18bc | 17.55±0.85ab | 29.11±1.64a | 15.59±1.75a | 16.45±1.00d | 17.38±1.05e |
| Immortality | 14.51±0.72bc | 15.57±4.48ab | 22.80±3.00a | 18.02±5.93a | 19.98±1.91bcd | 33.48±1.11a |
| White and Gold | 17.07±0.28bc | 18.36±1.78ab | 26.93±7.95a | 17.10±2.62a | 17.66±0.77cd | 19.85±2.13de |

(E)The Pro content of *I. germanica* leaves at different stress times (μg·g^-1^ FW)

| Pro content  Cultivars | Days of drought stress/ days | | | | | |
| --- | --- | --- | --- | --- | --- | --- |
|  | 0 | 6 | 12 | 18 | 24 | 30 |
| Cherry Garden | 175.25±73.35cd | 60.61±15.74cde | 465.18±94.79ab | 1112.98±378.94a | 143.85±27.59b | 258.15±47.35a |
| Tantara | 70.04±16.10d | 46.93±9.49de | 209.04±28.61cd | 868.75±159.22ab | 71.56±14.24c | 179.79±38.00ab |
| Blood Stone | 126.19±49.01d | 93.71±27.52bc | 556.40±44.57a | 947.92±73.68ab | 280.31±17.43b | 192.85±39.44ab |
| Music Box | 194.37±82.36bcd | 160.52±19.14a | 488.86±83.56a | 838.94±181.30ab | 256.73±20.19a | 282.13±56.76a |
| Memory of Harvest | 127.27±59.52d | 47.14±11.41e | 401.14±138.26ab | 1108.17±384.76a | 120.49±25.69bc | 189.83±22.29ab |
| Clarence | 102.02±22.22d | 88.93±22.51bcd | 140.61±37.85cd | 770.35±184.16ab | 141.03±22.82b | 187.25±49.23ab |
| X’Brassie | 173.09±58.79cd | 54.24±8.15de | 536.23±147.76a | 1201.12±350.28a | 136.54±49.45bc | 269.09±62.21a |
| Little Dream | 349.49±117.23ab | 80.33±12.75bcde | 298.33±46.38bc | 978.04±171.39ab | 131.04±13.22b | 131.90±6.09b |
| Immortality | 466.02±130.12a | 71.92±3.65bcde | 46.09±15.12d | 673.35±112.83ab | 96.30±17.85bc | 85.88±13.20b |
| White and Gold | 302.06±57.90bc | 107.73±14.64b | 153.18±47.54cd | 438.30±96.34b | 139.03±12.19b | 278.72±75.83a |

(F) The SP content of *I. germanica* leaves at different stress times (mg·g^-1^ FW)

| SP content  Cultivars | Days of drought stress/ days | | | | | |
| --- | --- | --- | --- | --- | --- | --- |
|  | 0 | 6 | 12 | 18 | 24 | 30 |
| Cherry Garden | 3.76±0.03a | 12.06±0.22a | 6.61±0.11a | 5.98±0.29ab | 5.827±0.29b | 6.42±0.16ab |
| Tantara | 3.71±0.28a | 11.61±0.57ab | 5.74±0.34abc | 6.13±0.80ab | 8.16±0.80a | 5.92±0.44abc |
| Blood Stone | 3.73±0.18a | 11.84±2.65a | 6.19±1.50ab | 6.30±1.35a | 5.79±0.46b | 6.31±0.84ab |
| Music Box | 3.54±0.27a | 11.43±2.28ab | 6.29±2.04ab | 5.94±0.70ab | 5.81±0.51b | 6.54±1.97a |
| Memory of Harvest | 2.45±0.69bc | 8.12±1.54d | 4.64±1.19cd | 5.06±0.84c | 4.98±1.03b | 5.42±0.26c |
| Clarence | 2.15±0.46c | 5.73±0.67e | 4.06±0.81d | 3.76±0.14d | 4.76±0.69b | 4.09±0.48d |
| X’Brassie | 3.72±0.13a | 11.08±0.76ab | 4.93±0.57cd | 6.03±0.32ab | 4.74±0.62b | 5.57±0.64bc |
| Little Dream | 3.74±0.14a | 10.82±1.22abc | 6.42±0.98a | 6.16±0.48a | 5.98±1.47b | 6.67±2.13a |
| Immortality | 3.32±0.54ab | 9.12±1.23cd | 5.48±0.60abc | 5.75±0.36abc | 5.43±0.43b | 6.40±0.467abc |
| White and Gold | 3.26±0.49ab | 9.72±1.40bcd | 5.16±0.21bcd | 5.28±0.53bc | 5.49±0.42b | 6.02±2.79abc |
