# Supplementary material for: Evaluation of drought resistance and transcriptome analysis for the identification of drought-responsive genes in Iris germanica
Source: Sci Rep. 2021 Aug 11;11:16308. doi: 10.1038/s41598-021-95633-z (PMC8358056; doi:10.1038/s41598-021-95633-z)
Supplement: Supplementary file 9 — Supplementary Table S5. [file 41598_2021_95633_MOESM9_ESM.docx]

| Unigene NO. | Description | Up or Down | Forward Primer | Reverse Primer |
| --- | --- | --- | --- | --- |
| Cluster-34938.174776 | Auxin response factor 19-like | Up | ACAACGTTACTCTGCATGCG | GCTTGTGTCACTTGCAGTCA |
| Cluster-34938.186959 | Auxin response factor 18 | Up | TGTTCGGGGATGAGGTTCAA | GGCATGTTCGATACCAGCTC |
| Cluster-34938.165591 | AP2 domain | Down | ATGTTTGCCTTGCCTTCGTT | AACCCAATCACAAGCTGCAG |
| Cluster-34938.157916 | WRKY transcription factor 4 | Down | TGACCATACTTCCGCCAGTT | TCCATCACACCTCTGCCATT |
| Cluster-34938.170167 | RINT-1 / TIP-1 family | Down | CTCTGCCTGAAAAGCTTGCA | AACACGTGAAACCAGAAGCT |
| Cluster-34938.158107 | E3 ubiquitin-protein ligase RFWD3 | Down | GAGGCCCAGGTATCACAGTT | TCCCACCAGAAGCTCCAATT |
| Cluster-34938.168662 | WRKY DNA -binding domain | Up | GCTCTGTTTGTTGTGCTCCA | CCTGCAATTAGTGGCGTACC |
| Cluster-34938.182173 | AP2 domain | Down | TTGCCCTTCGTGGTTTTGTT | GCAATCTCAAGTTCCCACGG |
| Cluster-34938.170411 | bZIP transcription factor | Up | CAACACCGTCAACAAGCTCA | GTGGAGGGGTAGCATCATCA |
| Cluster-34938.149407 | WRKY transcription factor 31 | Up | GCTGATGCCGGATGTTTGAT | TTTCAGGGCTTCACTCGTCT |
| *Tubulin* | ----- | ----- | TGGTTAGAAAGGAGGCAGAGAATTG | CAGTGTTCCCATACCAGATCCAGTC |

**Supplementary Table S5.** List of primer sequences used for RT-qPCR.
